# Transcriptome-Based Analysis of Tomato Genotypes Resistant to Bacterial Spot (*Xanthomonas perforans)* Race T4

**DOI:** 10.3390/ijms21114070

**Published:** 2020-06-06

**Authors:** Rui Shi, Dilip R. Panthee

**Affiliations:** 1 Department of Horticultural Science, Mountain Horticultural Crops Research & Extension Center, North Carolina State University, Mills River, NC 28759, USA; rshi@ncsu.edu; 2 Department of Crop and Soil Science, North Carolina State University, Raleigh, NC 27695-7620, USA

**Keywords:** bacterial spot, differentially expressed genes, RNA-seq, SNP/INDELs, tomato, *Xanthomonas perforans* race T4

## Abstract

Bacterial spot (BS) is one of the most devastating foliar bacterial diseases of tomato and is caused by multiple species of *Xanthomonas*. We performed the RNA sequencing (RNA-Seq) analysis of three tomato lines with different levels of resistance to *Xanthomonas perforans* race T4 to study the differentially expressed genes (DEGs) and transcript-based sequence variations. Analysis between inoculated and control samples revealed that resistant genotype *Solanum pimpinellifolium* accession PI 270443 had more DEGs (834), followed by susceptible genotype tomato (*S. lycopersicum* L) breeding line NC 714 (373), and intermediate genotype tomato breeding line NC 1CELBR (154). Gene ontology (GO) terms revealed that more GO terms (51) were enriched for upregulated DEGs in the resistant genotype PI 270443, and more downregulated DEGs (67) were enriched in the susceptible genotype NC 714. DEGs in the biotic stress pathway showed more upregulated biotic stress pathway DEGs (67) for PI 270443 compared to more downregulated DEGs (125) for the susceptible NC 714 genotype. Resistant genotype PI 270443 has three upregulated DEGs for pathogenesis-related (PR) proteins, and susceptible genotype NC 714 has one downregulated R gene. Sequence variations called from RNA-Seq reads against the reference genome of susceptible Heinz 1706 showed that chr11, which has multiple reported resistance quantitative trait loci (QTLs) to BS race T4, is identical between two resistant lines, PI 270443 and NC 1CELBR, suggesting that these two lines share the same resistance QTLs on this chromosome. Several loci for PR resistance proteins with sequence variation between the resistant and susceptible tomato lines were near the known *Rx4* resistance gene on chr11, and additional biotic stress associated DEGs near to the known *Rx4* resistance gene were also identified from the susceptible NC 714 line.

## 1. Introduction

Bacterial spot (BS) is a foliar disease of tomato caused by multiple species of *Xanthomonas*. There are four physiological races of *Xanthomonas,* including races T1 to T4, which are distributed throughout the world, particularly in warm and humid regions, and cause a significant yield loss every year. Association between races and species is classified as race T1 (*X. euvesicatoria*, *Xe*), race T2 (*X. vesicatoria*, *Xv*), race T3 and T4 (*X. perforens*, *Xp*), and no race designations (*X. gardneri*, *Xg)* [1,2].

Resistance to BS is both a monogenic and polygenic trait [2]. Multiple resistance genes and loci for BS have been identified in tomato and have been summarized by Pei et al. [3]. Hawaii 7998 is a differential genotype for identifying race T1, which includes *Xanthomonas* spp.—carrying the *avrRxv* gene [4]. Hawaii 7998 remains the most reliable source of resistance to race T1 [3,5], which is conferred by three independent loci (*rx1* and *rx2* on opposite arms of chromosome 1 and *rx3* on chromosome 5) and may be modified by three susceptible loci on chromosomes 3, 9, and 11 [6]. The dominant allele *Rx3* confers resistance in the field, explaining 41% of the phenotypic variation [7] and is considered to be the most effective locus for T1 resistance. It is not clear yet whether *rx3* and *Rx3* are alleles of the same gene or closely associated genes on the same chromosome [3].

Resistance to race T2 has been documented in Hawaii 7983, which expressed partial resistance over multiple seasons [8]. As *Xp* contains the major races (T3 and T4) that cause many annual epidemics in tomato growing regions [2,9], much work has been done to identify and breed resistance against this group of the *Xanthomonas*.

Race T3 resistance has been identified in several lines, including Hawaii 7981, and *Solanum pimpinellifolium* accessions PI 126932 and PI 128216, each conferring a hypersensitive response (HR) in the presence of the pathogenic expression of the *avrXv3* gene, and partial resistance in field assessments [10]. Race T4 resistance has been identified in LA 716 (*S. pennellii*), being conferred by *Xv4*. The previously mentioned line PI 114490 also showed strong resistance to race T4.

Mapping and characterization of loci help to develop resistant breeding lines. The quantitative trait loci (QTLs) associated with resistance to BS race T4 were identified in the populations derived from genotype Hawaii 7998 and PI 114490 and mapped to chromosome 11 and chromosome 3, respectively [11].

Resistance to BS is very complex, and fulfilling the introgression of resistance into the desirable genetic background is very challenging. Although the QTLs with the reasonably high level of *R^2^* value (29.4% and 4.8%, respectively, from chromosomes 11 and 3) to BS race T4 had been identified using QTL mapping method [11], its introgression into the breeding lines to achieve the desired level of BS resistance has not been obtained. In this context, it is reasonable to investigate the gene regulation network in more detail so that the resistance mechanism can be understood better. Differential gene expression analysis by conducting RNA sequencing (RNA-Seq) experiments has been used to understand such mechanisms in different species [12]. Recently, Du et al. [13] investigated the expression profiles of genes in response to BS race T3 infection and found that 78 genes were upregulated in PI 114490 (resistant parent), and 15 genes were upregulated in OH 88,119 (susceptible parent) six days after inoculation (dai). With information on gene expression in response to race T3 available, it is logical to investigate the genes specially expressed on exposure to BS race T4 in tomato, which has not been reported to date. With the availability of detailed annotation for most of the genes in the tomato genome, functional classification of the genes and pathway analysis is now much more convenient. A gene expression analysis approach using RNA-Seq analysis technology has been applied to unravel the gene function and defense mechanism in tomato, soybean, and several other plant species [13,14]. This approach is useful to identify the gene(s) associated with complex traits by comparing the detailed network of gene regulation between host and pathogens and eventually determining the phenotypic trait [12,15]. This approach is suitable to identify the gene network involved in conferring resistance to BS.

The available tomato genome sequence [16] also presents an opportunity to identify sequence polymorphism in the form of single nucleotide polymorphisms (SNPs), insert and deletion (INDELs) genome-wide, which can be developed into molecular markers for breeding program [17,18,19]. RNA-Seq data is a valuable resource for the detection of SNP/INDELs from gene transcripts, a subset of the whole genome sequence, but can be more relevant to functional analysis. For instance, to detect nonsynonymous SNP/INDELs, which lead to gene function alternation due to amino acid sequence change [18].

In North Carolina, *X. perforans* race T4 is the dominant race [20]. There are limited QTL analysis information and genetic dissection information available as of now. In this situation, we evaluated a few tomato breeding lines for BS resistance. Based on this screening, we selected three lines with a relatively good level of resistance and susceptibility to BS. These lines were used for transcriptome-based analysis in this study.

## 2. Results

### 2.1. Tomato Lines with Different Level of Resistance to BS

The resistance level of 30 tomato breeding lines was evaluated by inoculation with BS (*X. perforans*) race T4 in the greenhouse at Mountain Horticultural Crops Research and Extension Center, Mills River, NC, USA. Leaf samples of these lines were collected and frozen in liquid nitrogen 48 h after BS inoculation and then stored at −80 °C before RNA extraction and RNA-Seq analysis.

The resistance level of these 30 tomato lines, as shown by the area under disease progress curve (AUDPC) based on the data from greenhouse experiments, was used as a single indicator to select the genotype for disease resistance. We started scoring the plants 21 days after planting (dap) and continued until 35 dap. Among these tomato genotypes, accession PI 270443 was the most resistant line, followed by PI 114490, CLN-2413A, LA 2093, LA4277, and Fla. 7060_Xv4 with an AUDPC value of less than 280 (Table 1). Average disease incidence took place about 5.5 days after inoculation (dai) in LA2093, whereas it was 6 dai in PI 114490, LA4277, and Fla.7060_Xv4. In the case of PI 270443 and CLN2413A, the average disease incidence was 7 dai. Among the tomato lines, Heinz 1706 was the most susceptible line, followed by NC 714, NC 6 Grape, NC EBR7, and Hawaii 7981 with an AUDPC value of more than 608 (Table 1). The time for the first disease symptom appearance was not much different in the group of susceptible lines, which ranged from 5.5 to 6 days. Even in the wide array of lines, NC 1CELBR was intermediate in its response to the BS along with other lines, including NC 25P, NCEBR8, CLN-2418A, NC 161L, NC EBR6, Money Maker, LA2653, and NC123S. The level of disease development reported in terms of AUDPC in these lines ranged from 420 to 540 (Table 1). The disease developed very quickly in the susceptible lines, whereas the rate of disease development was very slow in the resistant lines. Hawaii 7998, which is widely used as a resistant line to BS, was very close to NC 1CELBR in AUDPC value, whereas NC 714 was the most susceptible line. 

**Figure 1:**
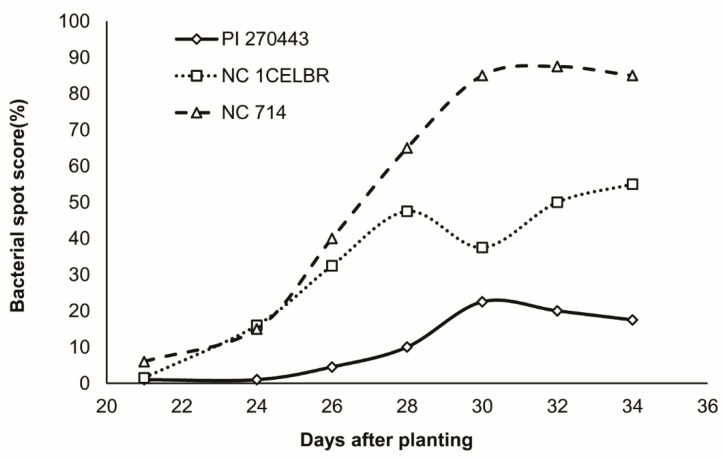
The resistance level of three selected tomato lines for BS.

Based on the results presented in Table 1, three tomato lines were selected to represent a different level of resistance to BS for RNA extraction and subsequent RNA-Seq analysis. These three lines were PI 270443, the most resistant tomato line, NC 714, one of the most susceptible tomato breeding lines, and NC 1CELBR, a medium resistance line (Table 1, Figure 1). Although Heinz 1706 is the most susceptible line in our list, it is a processing tomato, but our ultimate interest is to develop genomic resources using large-fruit fresh-market tomato breeding lines. Therefore, we selected NC 714 as the most susceptible lines instead of NC 5Grape, a small fruit tomato and Heinz 1706, a processing variety. NC 1CELBR is also a large-fruited fresh-market breeding line (Table 1, Figure 1).

### 2.2. RNA-Seq Library Information

To investigate the differences in transcriptome associated with the inoculation of BS race T4, 12 RNA-Seq libraries were constructed and sequenced with two biological replicates for three tomato lines (lines) inoculated with BS (IN) or without BS inoculation (CK). A total of 267 million reads were generated for these RNA-Seq libraries. These reads were processed to remove low-quality reads and then mapped to the Heinz 1706 genome assembly (SL3.0). The mapped RNA-Seq reads were quality filtered and resulted in a total of 234 million mapped reads (at least 6 million per library) for subsequent bioinformatics analysis (Table 2). The data of these RNA-Seq libraries have been deposited in NCBI’s Gene Expression Omnibus [21] and are accessible through GEO Series accession number GSE135232.

**Figure 2:**
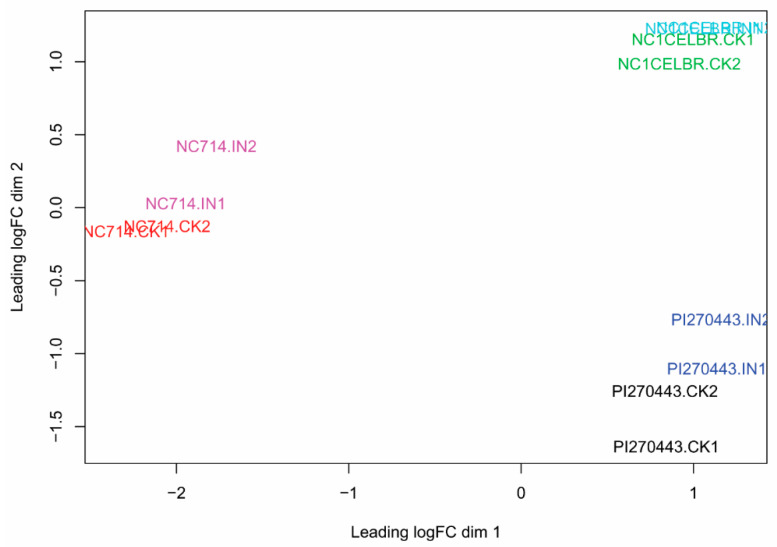
Multidimensional scaling plot for the relationship of each RNA-Seq library. Distance between libraries was calculated as the leading log fold change for biological variation between libraries [23]. The label of “IN” represents BS-inoculated samples, and “CK” represents a control sample without inoculation of BS.

The first step of our analysis was to check the relationship of all RNA-Seq libraries based on variation distance calculated from RNA-Seq read counts for annotated genes using the plotMDS function of the Edge R package [22]. Ideally, replicated samples from the same group should cluster together in the plot, while samples from different sample groups form separate clusters [22]. In this plot, we found that samples of each tomato lines are grouped. However, RNA-Seq libraries for uninoculated (CK) and inoculated (IN) samples separated slightly from each other for the same tomato line (Figure 2). This plot pattern also indicated that gene expression variation between lines was more significant than BS inoculation treatment. Based on such expression profiles, we adopted the strategy of analyzing genes by comparing expression changes between treatment (control and inoculated) samples within the same genotype first, and then compare inoculation-induced DEGs between different tomato lines.

### 2.3. DEGs Induced by BS Inoculation

**Figure 3:**
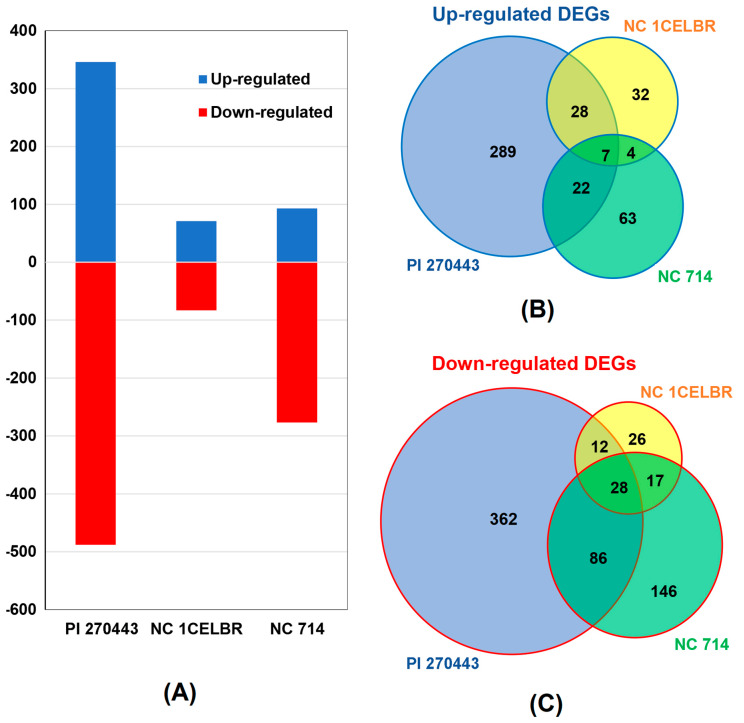
DEGs in three tomato lines induced by BS inoculation. (**A**) The number of upregulated (blue bar) and downregulated DEGs (red bar, with the label of “-”) in tomato lines with respect to the inoculation of *X. perforans* race T4. (**B**,**C**) Venn diagram showing the upregulated (blue outlined) and downregulated (red outlined) differentially expressed genes (DEGs) in three tomato lines PI 270443, NC 1CELBR, and NC 714. An entire list of differentially expressed genes (DEGs) in all three tomato lines is provided as Supplementary Material (Table S1).

Differentially expressed genes (DEGs) were screened between control and inoculated tomatoes for each breeding lines based on a 2-fold change of transcript abundance and FDR <0.05. A total of 1161 differentially expressed genes without overlap were identified from this study. Among them, PI 270443 had 834 (346 upregulated, and 488 downregulated), NC 1CELBR had 154 (71 upregulated, 83 downregulated), and NC 714 had 373 (93 upregulated, 277 downregulated) DEGs between control and inoculated samples, respectively (Figure 3A, Table S1).

Overall, there were 445 upregulated genes and 677 downregulated genes without overlap in the three lines. Among them, only 35 genes were common (seven upregulated and 28 downregulated) among all three tomato lines, and these genes represent a common reaction to BS inoculation (Figure 3B). More combinations, as shown in Figure 3B, also indicated that PI 270443 has 289 up and 362 downregulated DEGs specific to PI 270443 over two other lines, and these genes might be associated with strong resistance reaction to pathogen attacks. In comparison, NC 714 had 63 and 146 up and downregulated DEGs specific to NC 714 over two other lines, and these DEGs might be associated with several susceptible reactions to pathogen attack. DEGs specific to NC 1CELBR might be more complicated to interpret, but its common DEGs with PI 270443 might be more helpful for resistance, and DEGs shared with NC 714 may be associated with susceptibility.

### 2.4. Gene Ontology Term Enrichment from BS Inoculation-Induced DEGs

**Figure 4:**
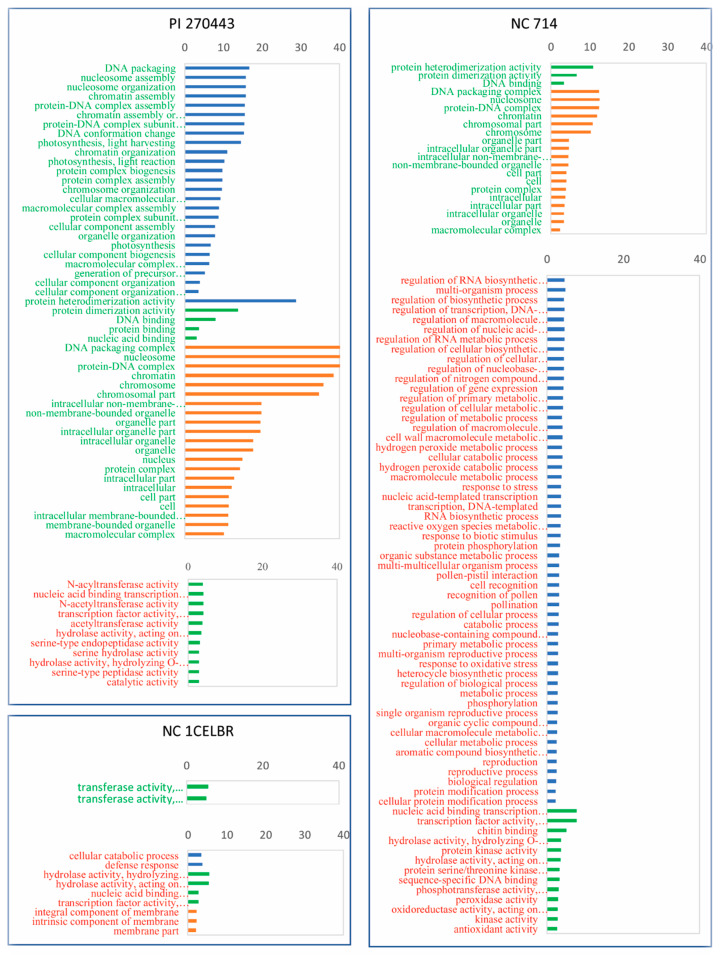
GO terms enriched for DEGs in different tomato lines after BS inoculation. Labels in green and red represent upregulated and downregulated DEGs, respectively. Bars colored in blue, green, and orange represent a biological process, cellular component, and molecular function GO category, respectively. Scale is log10 (1/*p* value)**.**

Functional analysis of DEGs for different tomato lines was conducted based on gene ontology (GO) enrichment analysis using AgriGO based on tomato ITAG3.2 version annotation and background. We expected that some GO terms were enriched (over-represented) after BS inoculation, and provide clues to understand the reaction of BS inoculation for different tomato lines. Based on this analysis, we found more GO terms enriched for upregulated DEGs in resistant genotype PI 270443, and more GO terms were enriched for downregulated DEGs in susceptible genotype NC 714 (Figure 4, Table S2).

In detail, there were 51 GO terms enriched among the 346 upregulated DEGs from PI 270443. Among the enriched GO terms, the important GO terms included protein binding, cell parts, cell, intracellular, intracellular part, intracellular organelle, organelle, nucleic acid binding, DNA binding, and protein complex (Table S2). Additional important GO enriched terms were intracellular non-membrane-bounded organelle, non-membrane-bounded organelle, and macromolecular complex, among others.

For 488 downregulated DEGs from PI 270443, there were 11 GO enriched terms with a relatively low level of enrichment, and all these ontologies are assigned as molecular functions. The important GO terms were catalytic activity, nucleic acid binding transcription factor activity, transcription factor activity, sequence-specific DNA binding, hydrolase activity, acting on glycosyl bonds, hydrolase activity, hydrolyzing O-glycosyl compounds, serine hydrolase activity, and serine-type peptidase activity, among others (Figure 4, Table S2).

There were only two GO terms enriched for NC 1CELBR among its 71 upregulated DEGs, which are transferase activity for hexosyl groups and glycosyl groups. Nine GO terms were enriched for 81 downregulated DEGs from NC 1CELBR, including membrane part, hydrolase activity, hydrolyzing O-glycosyl compounds, hydrolase activity (acting on glycosyl bonds, an integral component of the membrane), cellular catabolic process, and defense response (Figure 4, Table S2).

For the 96 upregulated DEGs from NC 714, there were 21 GO enriched terms. The significantly enriched terms were associated with cellular components such as DNA and chromosome (Table S2). For the 277 downregulated DEGs from NC 714, there were 67 GO terms enriched, including response to stress (GO:0006950), and biotic stimulus (GO:0009607), protein kinase activity, peroxidase activity, antioxidant activity, response to biotic stimulus, cell recognition, chitin-binding, protein phosphorylation, regulation of the cellular process, cellular metabolic process, and response to oxidative stress, among others (Figure 4, Table S2).

Overall, there were several enriched GO terms for upregulated DEGs from PI 270443, which might be associated with the resistance of this genotype. Contrary to this, more enriched GO terms for the downregulated DEGs of NC 714 might be associated with the susceptibility of NC 714. It should be noted that there was no overlap of GO terms for all upregulated DEGs from these three lines, but all 21 GO terms enriched in the upregulated DEGs of NC 714 overlapped with the GO enrichments from PI 270443. The lack of GO overlap between NC 1CELBR upregulated DEGs and the other two lines might be associated with few DEGs identified in this genotype. There were four GO terms enriched for downregulated DEGs for all these three lines, which might refer to a common reaction to BS. These GO terms include nucleic acid binding transcription factor activity, transcription factor activity, sequence-specific DNA binding hydrolase activity, and acting on glycosyl bonds hydrolase activity, hydrolyzing O-glycosyl compounds.

### 2.5. BS Inoculation Induced DEGs in the Biotic Stress Pathway

**Figure 5:**
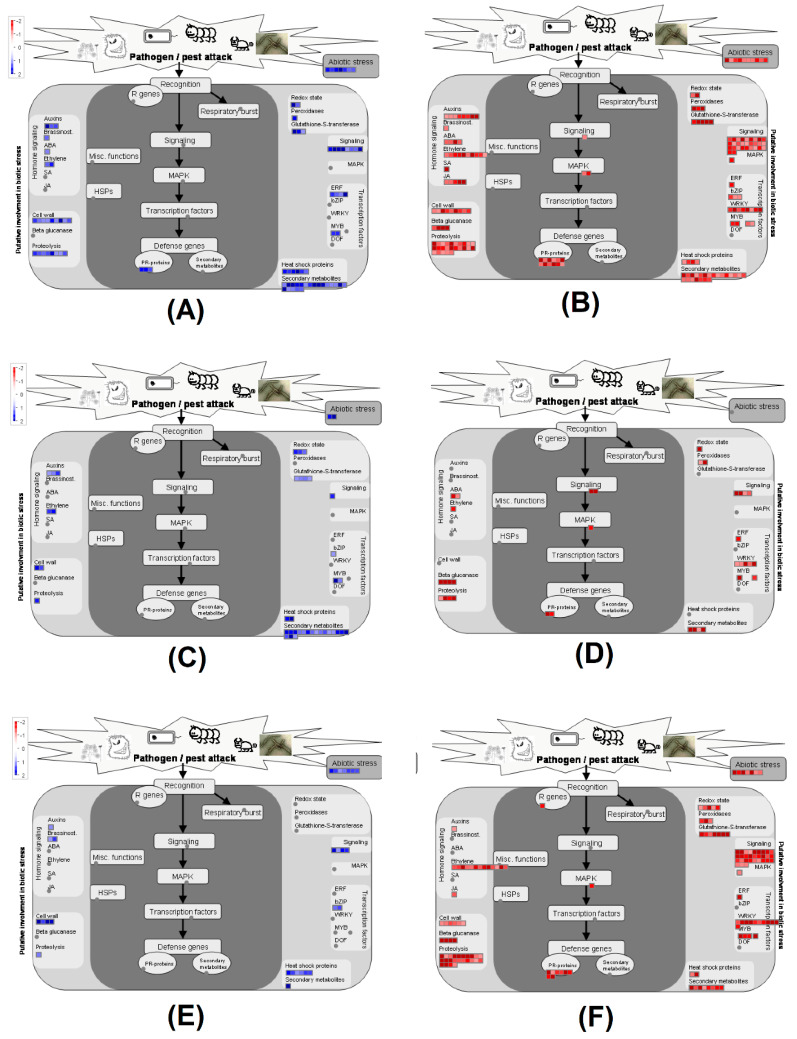
BS inoculation-induced DEGs in the biotic pathway for different tomato lines. Illustration of (**A**) PI 270443 upregulated DEGs (blue color) (**B**) PI 270443 downregulated DEGs (red color). (**C**) NC 1CELBR upregulated DEGs (blue color) (**D**) NC 1CELBR downregulated DEGs (red color). (**E**) NC 714 upregulated DEGs (blue color) (**F**) NC 714 downregulated DEGs (red color) in the biotic stress pathway via MapMan.

To understand the responses of these tomato lines to BS infection, MapMan was used to classify the DEGs of these tomato lines based on the more comprehensive annotation of tomato genes as described in the methods section. This analysis was conducted to decipher the involvement of DEGs in various cellular processes using the MapMan software. In Figure 5, we present the distribution of DEGs in the biotic stress pathway, which is associated with disease resistance and development.

Overall, highly resistant genotype PI 270443 had more upregulated DEGs (67) in the biotic stress pathway (Figure 5A), followed by 34 upregulated DEG in NC 1CELBR (Figure 5C), and 21 upregulated DEGs in NC714 (Figure 5E). On the other hand, upregulated DEGs in PI 270443 had more bins (catalogs defined for genes relating to functional processes in MapMan) and more DEGs in each bin as well. For instance, PI 270443 had three upregulated PR-protein genes in the core of biotic stress pathway (dark area in Figure 5A) and had DEGs associated with ABA and peroxidase, which were not present in other two lines. There were also more DEGs associated with the cell wall, proteolysis, signaling, Erythroblast transformation specific domain-containing transcription factor (ERF), and secondary metabolites for PI 270443 than in the other two lines. Although the overall DEGs and enriched GO terms for NC 1CELBR seemed underestimated in our analysis, this medium resistant tomato line still had more upregulated DEGs in biotic stress pathway than that of NC 714. The DEGs were associated with auxins, ethylene, redox-associated genes, glutathione-S-transferase, heat shock proteins, and secondary metabolites.

Regarding downregulated DEGs in this biotic stress pathway, PI 270443 had 154 DEGs (Figure 5B), followed by 126 in NC 714 (Figure 5F), and 33 in NC 1CELBR (Figure 5D). Regarding each catalog, the pattern of PI 270443 and NC 714 was similar in number, but NC 1CELBR had few DEGs in each bin (Figure 5D). One noticeable pattern was that one DEG (solyc12g097000) defined as R gene, which is essential for disease resistance, was downregulated in NC 714 (Figure 5F), and no such DEG was found in the biotic stress pathway for the other two lines (Figure 5B,D).

Table 3 presents the overlapping DEGs in the biotic stress pathway for these tomato lines. It shows that among 67 DEGs upregulated in PI 270443, only two were commonly upregulated DEGs in all three lines, which might be associated with a common reaction to the inoculation of BS race T4. There were 16 DEGs upregulated in both PI 270443 and NC 1CELBR, which are resistant to BS. One noticeable feature was that half of them are involved in the secondary metabolism of flavonoids. Furthermore, the *CCoAOMT* gene, which was reported to be disease-resistance-associated in maize [24,25,26,27,28,29,30], was found as the common upregulated DEG in these two resistant tomato lines. Regarding upregulated DEGs specific to PI 270443, some groups of genes were overrepresented, including those associated with cell wall cellulose synthesis, APETALA2/ethylene-responsive element binding protein family, protein degradation related, and stress-related genes. On the other hand, among the 125 downregulated DEGs in NC 714, which might be associated with susceptibility to BS race T4, there were 15 commonly downregulated genes in all three lines, including two secondary metabolisms, lignin genes *C3H* and *F5H*, and three genes encoding *β*-1,3 glucan endo-1,3-*β*-glucosidase. These DEGs, we believe, are common reactions to the inoculation of BS. Downregulated DEGs specific to NC 714 included three *CCoAOMT* genes, five WRKY domain transcription factor genes, and five genes related to protein degradation/ubiquitin in the list of the common downregulated gene for PI 270443 and NC 714 (Table 3).

### 2.6. BS Inoculation-Induced DEGs in Other Immunity Levels.

We attempted to evaluate the possible involvement of genes in the level of plant immunity based on DEGs associated with effector-triggered immunity (ETI) and pattern-triggered immunity (PTI) identified by Pombo et al. [31]. They reported Epk1 as a novel protein kinase for the induction of host response for the recognition of three beneficial bacterial effectors. We found a pattern of ETI and PTI associated with the level of resistance in three lines. For instance, there were more ETI-specific genes found in upregulated DEGs of resistance genotype. PI 270443 had 30 upregulated DEGs specific to ETI, whereas NC 1CELBR and NC 714 had 12 and 11 genes, respectively. Regarding PTI-specific genes, only one (Solyc07g008440) gene was identified among upregulated DEGs from PI 270443, but no PTI-specific gene was found in NC 1CELBR and NC 714. Genes commonly associated with ETI and PTI (ETI-PTI) which might be involved in a common resistance pathway to pathogens can be found in both PI 270443 (7) and NC 1CELBR (6), the two lines with strong and medium resistance to BS, but there were no genes in NC 714, which is the most susceptible to BS (Table 4).

There were fewer ETI/PTI-associated DEGs induced by BS inoculation in our study compared to Pombo et al. [31]. For instance, Pombo et al. [31] found 2805/4274 DEGs from 6 h after inoculation (hai) using the vacuum-infiltration method on leaf tissue. In contrast, we found 57/139, 18/38, and 26/105 DEGs in PI270443, NC 1CEBEL, and NC714 induced by inoculation using the spray method, respectively. These numbers are comparable reported by Du et al. [13] from their RNA-Seq analysis based on 6 hai spray-inoculated samples.

### 2.7. Transcriptome-Based Sequence Variation in Three Selected Tomato Lines

The SNP molecular marker analysis was conducted based on a sequence of RNA-Seq reads mapped to reference genome sequence of tomato Heinz 1706 (SL3.0), which is the most susceptible to BS race T4. Overall, a total of 23,253 SNP/INDELs sites were called in PI 270443, NC 1CELBR, and NC 714 against Heinz 1706. Among these SNP/INDELs, 18,238 could be located within the sequence of 6676 gene models (annotation version ITAG3.2). Distribution of these SNP/INDELs and associated genes on the individual chromosome are listed in Table 5. As SNP/INDELs or related genes in individual tomato lines, these were defined as a homo form of alternative sequence to Heinz 1706, i.e., GT:1/1 in the genotype section of the VAR file. More detailed SNP/INDELs raw information can also be found in Table S3.

These results showed that in NC 714, the most susceptible tomato breeding line had more SNP/INDELs against the reference genome of similar susceptible Heinz 1706. In contrast, the most resistant PI 270443, which is expected to be different from Heinz 1706 (susceptible to BS), had fewer SNP/INDELs. The situation of the individual chromosome was different. For instance, chr11 and chr12 of NC 714 had relatively fewer SNP/INDELs and associated genes than Heinz 1706, but PI 270443 and NC 1CELBR had more SNP/INDELs and associated genes, respectively.

**Figure 6:**
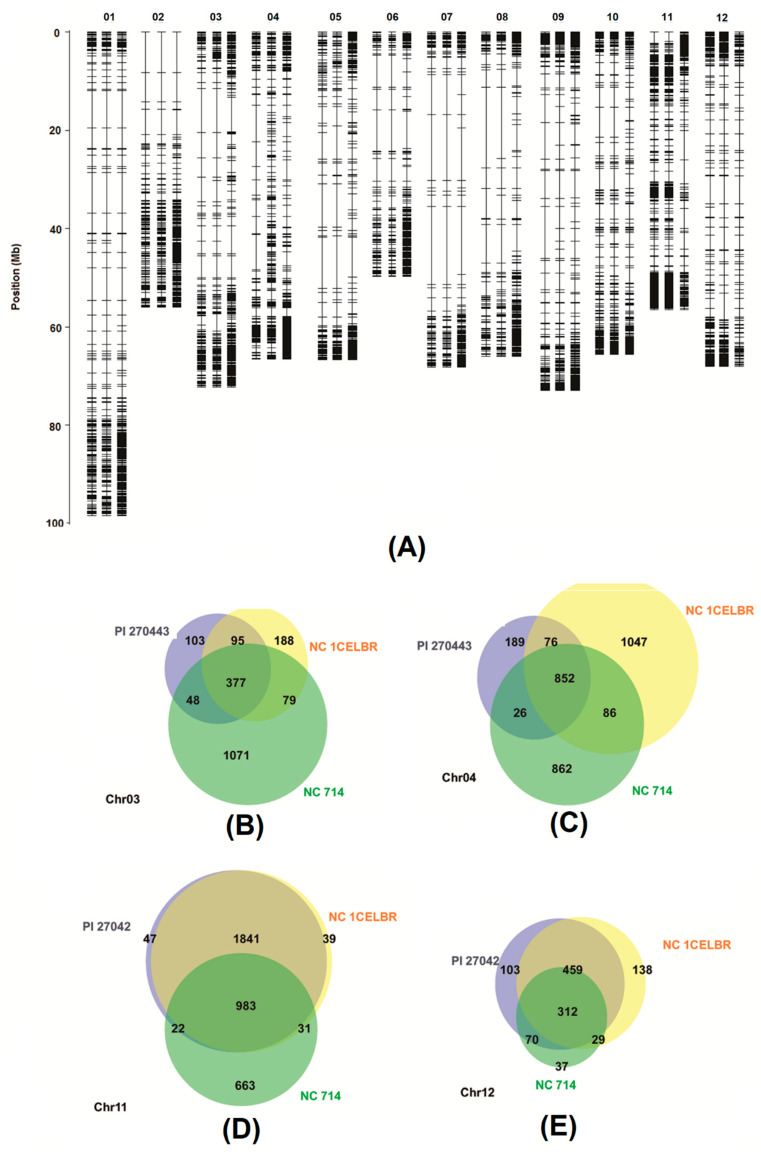
Analysis of sequence variation identified from transcriptomes of three tomato lines. (**A**) Distribution of genes with SNP/INDELs on different chromosomes. (**B**–**E**) are Venn diagrams for overlaying SNP/INDELs identified from three tomato lines for chromosome 3, 4, 11, and 12 (labeled as chr03, chr04, chr11, and chr12).

On further evaluation of the distribution of sequence variation, as shown in the locations of genes with SNP/INDELs on each chromosome (Figure 6A), we noticed that the patterns of genes with SNP/INDELs on chr11 SNP/INDELs from PI 270443 and NC 1CELBR were almost the same, which can be further confirmed by comparison of SNP/INDELs between these two lines, resulting in over 95% identity on chr11 (Figure 6D). One critical insight of this pattern was that chr11 has resistance QTLs, as identified in the previous study, and these QTLs contribute 25% resistance to race T4 [11]. This pattern suggested that QTLs on chr11 might contribute to resistance of PI 270443 and NC 1CELBR.

In addition to a similar sequence variation pattern on chr11, which has resistance QTL as reported before [11], these two lines also share some common sequence variation on chr12 (Figure 6D), which has QTLs related to susceptibility to race T4 of BS [11]. Therefore, such common sequence variation in PI 270443 and NC 1CELBR against the reference genome of susceptible Heinz 1706 might be associated with reduced susceptible QTLs to the BS. SNP/INDELs on other chromosomes in these three lines were more diverse, such as chr3 and chr4, as shown in Figure 6B,C, even though it was reported that chr3 has resistance QTL to BS [11].

### 2.8. Analysis Combined with Transcriptome-Based Sequence Variation and Biotic Stress-Associated DEGs in Three Selected Tomato Lines

To further explore the application of RNA-Seq analysis in gene response to BS resistance, we checked all DEGs or sequence variation of genes with biotic stress pathway or putative involvement in disease resistance from chr11 (Table 6). Based on the table, it seems that most of chr11 (from 5 Mb to end) of PI 470443 and NC 1CELBR are identical, but different from NC 714 in terms of SNP/INDELs distribution. However, there was no apparent association between SNP/INDELs with upregulated DEGs with biotic stress pathways in this region. For instance, many genes had identical SNP/INDELs to PI 470443 and NC 1CELBR, but were different from NC 714. However, none of the upregulated DEGs in both PI 470443 and NC 1CELBR had SNP/INDELs, except one upregulated DEGs associated with disease resistance (solyc11g066580, secondary metabolism, flavonoids, Dihydroflavonols, flavonoid 3’’-monooxygenase in biotic pathway associated). In a reverse direction, we found one downregulated DEG (solyc11g020230, signaling. receptor kinases.crinkly like) and two upregulated DEGs (solyc11g066060, stress.abiotic.heat; and solyc11g069960, signaling. receptor kinases.leucine rich repeat III.) in NC 714 induced by BS. These genes are not DEGs in the other two lines with resistance to BS. All these genes contain identical SNPs in PI 470443 and NC 1CELBR, but not in NC 714. Therefore, these genes may be associated with BS resistance.

## 3. Discussion

The primary objective of this study was to determine and identify the unique DEGs in response to the BS race T4 so that we can identify the specific genes associated with BS resistance in tomato. It has been a challenge to determine the resistance genes, and hence to introgress the resistance into the breeding lines with desirable fruit quality in tomato despite many efforts towards the identification of QTL associated with BS resistance. In this study, the response of tomato lines to BS race T4 was as expected. For example, PI 114490, which is resistant to all races of *Xanthomonas*, also had less disease severity.

On the other hand, NC 714, which does not have any disease resistance to any race of *Xanthomonas,* was found to develop severe disease. An exciting aspect of this study was that PI 270443 had less disease than PI 114490. In several papers, PI 114490 has been reported to have resistance to all available races [10,11,32,33,34,35]. In our study, however, a genotype more resistant than PI 114490 is available, and the genes identified from the genotype may provide more robust information towards the identification of resistance genes. It should be noted that the race of BS reported from NC is race T4 [20]. This indicated that the source of resistance might be associated with a single race. This will still be a beneficial material to address this critical issue. Race T4 is widely distributed in FL and NC tomato-growing regions [20].

In exploring RNA-Seq library relationships by using plotting analysis, we found that the expression variation between different tomato lines was much bigger than inoculation treatment within the same tomato genotype based on calculated variation distance. Such a pattern may be associated with the genomic background of tomato lines. For instance, PI270443 (*S. pimpinellifolium*) is a wild species of tomato, and the tomato breeding line NC 1CELBR contains the genome segment of *S. pimpinellifolium*. Therefore, heterogeneous expression profile patterns between these tomato lines are expected. Genes in tomato lines with the *S. pimpinellifolium* genome are more diverse in sequence than tomato breeding lines. Such sequence diversity can lead to the underestimation of expression of wild tomato genes based on RNA-Seq read alignment of wild tomato genes to the Heinz 1706 tomato reference genome assembly. To overcome these effects in gene expression analysis, we adopted the strategy of comparing gene expression within the same line for screening BS inoculation-induced DEGs and then analyzing the resultant genes between different lines.

RNA-Seq analysis by this approach successfully identified DEGs associated with critical biological and cellular processes. While some DEGs are unique to the *Xanthomonas* race T4, others are already reported either with a plant defense system or with stress tolerance. In most of the gene expression or RNA-Seq studies, many pathways are involved, although biotic stress pathways were primarily focused on specific disease resistance-associated genes.

As expected, there was a significant difference between inoculated and control breeding lines for DEGs when evaluated based on RNA-Seq analysis. Du et al. [13] performed gene expression analysis using race T3 of *X. perforans* in tomato and identified more DEGs in resistant line PI 114490 than in susceptible line OH88119, and identified different sets of genes associated with cellular and molecular processes. Our analysis revealed that resistant accession PI 270443 had more DEGs compared to susceptible line NC 714, but NC 1CELBR, with a medium level of resistance, had fewer DEGs induced by inoculation of BS race T4. However, when we closely evaluated the upregulated DEGs in the biotic stress pathway, we identified more biotic stress-associated DEGs in NC 1CELBR than in NC 714, even though the total number of upregulated DEGs or enriched GO terms in NC 1CELBR were much less than that in NC 714.

All identified DEGs are valuable resources for the evaluation of plant reaction to BS infection, and GO term enrichment is the most adopted strategy to show the overall response based on over-representation. However, in this study, we also adopted MapMan-based analysis to identify genes associated with disease resistance. Our results showed a noticeable feature of a higher number of upregulated genes induced by the inoculation of BS in the resistant tomato line and a more significant number of downregulated genes found in the susceptible line after *X. perforans* race T4 inoculation. We tend to think that such patterns would provide an excellent opportunity to find resistance-related genes in resistant genotypes and genes associated with disease development in susceptible genotypes. For instance, in the core genes of the biotic stress pathway, we found that only resistant accession PI 270443 had three upregulated PR genes (loci solyc09g092300, solyc08g081790, and solyc09g092310) associated with inoculation of BS race T4. On the other hand, only the susceptible NC 714 line had a downregulated R gene (locus solyc12g097000) among the three lines. These genes were not reported in the study conducted by Du et al. [13]. A possible reason for this is that we analyzed samples collected two days after inoculation (48 hai), which is different from the condition of samples collected by Du et al. [13] after 6 hai and 6 dai. Additionally, the genetic background of their susceptible variety is substantially different from the tomato lines we analyzed.

Regarding the other bins of putative genes involved in the biotic stress pathway, we identified genes for glycosyl hydrolases (GH). The GHs comprise a large assembly of enzymes that hydrolyze the glycosidic bond between carbohydrates or between carbohydrates and noncarbohydrate moieties. GHs are grouped into various families based on amino acid sequence similarities. These proteins perform diverse functions in both plants and microbes. Many pathogenesis-related (PR) proteins belong to the GH group. GH family 17 and families 18 and 19, which contain β-1,3 glucanases and chitinases, respectively, form an essential part of the defense arsenal of plants against fungal pathogens [36,37,38].

We also found many genes associated with secondary metabolites involved in the reaction to inoculation of BS. For instance, in addition to many upregulated genes for secondary metabolites of flavonoids, we identified that *CCoAMOT* (solyc09g082660) was upregulated in PI 270443 after inoculation, and several *CCoAMOTs* (solyc02g093230, solyc02g093250, and solyc02g093270) were downregulated in NC 714 only. It should be mentioned that *CCoAMOT* has been reported as a gene involved in disease resistance [38,39,40]. On the other hand, *C3H* (solyc10g078220) and *F5H* (solyc12g042480) for lignin biosynthesis were downregulated in all tomato lines, which might be a common reaction to the inoculation of BS.

Genes with regulation functions involve more complex interactions. They need more power to analyze, but we still found that after inoculation of *Xanthomonas* race T4, there were more ethylene-responsive transcript factor genes upregulated in resistant PI 270443, and many *WRKY*s, on the other hand, were downregulated in susceptible genotype NC 714. Such reaction seems to be different from that reported by Du et al. [13], since *WRKY*s, including Solyc03g116890, Solyc04g051690, Solyc06g066370, and Solyc08g082110, were upregulated in both resistance line PI 114490 and susceptible line OH 88,119 after 6 hai, but downregulated 6 dai in these two lines in the BS race T3 inoculation experiment. This is possibly due to the different inoculation time of tomato samples with BS.

The BS inoculation-induced DEGs can also be interpreted via two interlinked layers of immunity to pathogen assault in the plant. One is pattern-triggered immunity (PTI) by detecting features of pathogen-associated molecular patterns (PAMPs). Then, induced expression of mitogen-activated protein kinase (MAPK) leads to further reaction to the pathogen. Another layer relies on the detection of the effector associated with the pathogen, and is therefore termed as effector-triggered immunity (ETI), usually associated with localized programmed cell death (PCD) as the hypersensitive response (HR) to pathogen invasion [41,42].

The identified ETI/PTI-associated DEGs induced by BS inoculation in our study are less represented in the ETI/PTI gene list than found by Pombo et al. [31]. We believe that this phenomenon is associated with the pathogen inoculation method. For instance, vacuum infiltration is more efficient in forcing pathogen into the host tissue and inducing significant reactions. While in spray inoculation, fewer host cells of the leaves are infected due to the physical and chemical barriers on the leaf surface to block the pathogen invasion [13]. In addition, we suspected that vacuum-filtration-induced reaction to pathogen invasion is more likely a localized defense response, and spray inoculation might involve a nonlocalized system acquired resistance (SAR) reaction in cells near infected cells, and therefore, the overall transcriptome-based reaction is less representative in vacuum filtrated samples.

Although the number of identified ETI/PTI DEGs are limited in this study, this analysis still provides clues towards the overall immunity pattern to BS. Induction of ETI-specific DEGs was identified in all tested tomato lines, while PTI-specific DEGs were only found in PI 470443, the genotype with strong resistance to BS, and common ETI/PTI DEGs were be identified in the PI 270443 and NC 1CELBR genotypes with resistance to BS, but not in the susceptible NC 714 genotype.

DEG analysis can reveal overall functions specific to the biotic stress pathway for genes associated with disease resistance and susceptibility processes. Sequence variation within expressed genes can also contribute to disease resistance even if they are not DEGs. Sequence variation in coding regions can alter the function of genes, and thus may affect disease resistance. For instance, the sequence variation in *Rx4* was found to be associated with resistance to race T3 BS in tomato [3]. Therefore, we have taken advantage of transcriptome information in this study to call SNP/INDELs and hope to find genes with sequence variation between BS-resistant and susceptible tomato lines.

Our results showed more SNP/INDELs in NC 714 against Heinz 1706, suggesting a more diverse genetic background between these two BS-susceptible lines. However, such sequence variation on chr11 was small, which suggested that both lines might lack resistance genes to BS from wild tomato at this chromosome. The chr11 of PI 270443 and NC 1CELBR were identical based on SNP/INDELs distribution, but different from that of NC 714 and Heinz 1706. This suggested that chr11 contributes to the resistance of tomato lines to BS. For instance, chr11 was reported to have QTLs contributing ~(*R^2^* = 29.4%) resistance to BS [11]. Chromosome 3 ~(*R^2^* = 4.3%), which has been reported to contain BS resistance elements, did not show such a pattern in the two resistant tomato lines, and might explain the different resistance levels in PI 270443 and NC 1CEBLR to BS infection. To focus more on BS race T4 resistance genes, we feel that it would be relevant to specifically concentrate efforts on chr11.

Line PI 128216, derived from *S. pimpinellifolium,* has been reported to carry the gene *Rx4* located on chromosome 11, which confers hypersensitivity and field resistance to race T3. An NBS-LRR class of resistance genes was fine-mapped near a 45.1 kb region between the pcc17 and pcc14 molecular markers. Six SNPs and one INDEL were also found in this region, which was found to be useful for the marker-assisted selection (MAS) of this gene [3].

The recessive genes, *rx1* and *rx2*, conferring resistance to race T1, were derived from Hawaii 7998 and mapped onto chr1, whereas *Rx3* was mapped onto chr3 [4]. Modifying susceptible alleles have been reported from chr3, 5, 9, and 11. The genes *avrXv3, Xv3/avrXv3*, and *Xv3/Rx4* derived from Hawaii 7981 and PI 126932, respectively, were located on chr11, conferring resistance to race T3 [43,44].

By blasting the published cloned *Rx4* candidate transcript sequence for *S. lycopersicum* lines Hawaii 7981, OH88119, and *S. pimpinellifolium* PI 128216PI 128216 (JF743044.1, JF743043.1, and JF743045.1, respectively), we found that the candidate *Rx4* gene is a homolog of solyc11g069020, for which we identified no SNP/INDELs in this study (Table 6), possibly due to their low expression levels in the tissues tested. On the other hand, we found multiple PR genes around this *Rx4* candidate gene (<2 Mb, Table 6), and these PR genes have SNP/INDELs specific to the resistant tomato line PI 270443 and medium resistant line NC 1CELBR compared to susceptible lines NC 714 and Heinz 1706 (Table 5). Although these PR genes are not identified as BS-induced DEGs in these two lines, they might still function diversely from their homologs in susceptible tomato plants, and might function as multiple loci for resistance to BS race T4.

RNA-Seq-based analysis usually focuses on gene expression profile, such as DEG identification. Here, we tried to combine it with transcriptome-based sequence variation to explore the possible reason for different reactions of tested tomato lines to BS inoculation. We illustrated a simple case by focusing on chr11. This chromosome seems highly similar in the two resistant tomato lines. Therefore, any genes with sequence variation and DEGs between the two resistant lines and the susceptible line are of great interest for future analysis. Using this comparison, we found some DEGs of interest. For instance, we identified some DEGs that are putatively involved in biotic stress pathways from chr11 in susceptible tomato line NC 714, while these genes were not differentially expressed in resistant lines PI 27043 and NC 1CELBR. On the other hand, these genes contain SNPs in resistant lines PI 270443 and NC 1CELBR, but no sequence variation was detected in NC714 (Table 6) or Heinz 1706. Based on this data, we suggest that these genes might be responsible for the sensitive response of NC 714. Two of these DEGs are located at 50.2 Mb and 54.9 Mb of chr11, respectively, near the location of the *Rx4* homolog (53.9 Mb).

## 4. Materials and Methods

### 4.1. Plant Materials

Thirty tomato lines grown in a greenhouse were evaluated for their resistance to bacterial spot (*X. perforans*). These tomato lines were sown in 4P soil mixture (Fafard^®^, Agawam, MA, USA) in 24-cell trays in the greenhouse at the Mountain Horticultural Crops Research & Extension Center, Mills River, NC, USA. Six plants per genotype were planted in duplicate in a completely randomized design. Plants were fertilized using a mixture of fertilizer containing a ratio of 20:20:20 of nitrogen, phosphorus, and potassium, respectively. Standard greenhouse treatments for insects and fungal disease management were used, but copper was not applied to control bacterial diseases.

Based on the disease score, three tomato lines (*S. pimpinellifolium* L. accession PI 270443, and *S. lycopersicum* L. breeding lines NC 1CELBR, and NC 714) were selected as tested lines to be used for the RNA-Seq analysis. PI 270443, a small-fruited tomato, was found to have the least level of BS and was used as the resistant genotype in this study. NC 714 is a large-fruited tomato breeding line with excellent horticultural traits developed in a NC State breeding program [45]. It developed the most BS disease. NC 1CELBR is also a large-fruited, late blight-resistant tomato breeding line developed from a NC State University tomato breeding program [46]. Late blight resistance comes from one of the *S. pimpinellifolium* (LA3707) lines. It had a medium level of BS disease resistance and was used as a tomato line in this study.

### 4.2. Bacterial Spot Inoculation and Disease Evaluation

Plants were artificially inoculated with Isolate 9 of *X. perforans*, which was found to be extremely virulent to many tomato cultivars. This is a field isolate collected from infected tissue of a tomato plant in western NC and characterized as *X. perforans* race T4 using differential tomato lines [5,47,48,49] by Dr. Jefferey B. Jones’ lab, University of Florida, Gainesville, Florida. The strain was maintained in pure culture and stored at −80 °C. The isolate was grown on yeast dextrose chalk (YDC) agar medium [50] at 28 °C for 24–48 h and was then overlaid with sterile distilled water. The bacteria were dislodged from the plates, and the resulting bacterial suspensions were pooled in a sterile glass container. The suspension was standardized by determining its optical density at 600 nm using an LKB Biochrom Ultrospec II Spectrophotometer (American Laboratory Training, East lyme, CT, USA) and diluted as needed to obtain an OD_600_ of 0.3 (approximately 2–5 × 10^8^ CFU/mL). Diluted cells were immediately used for inoculations.

For greenhouse inoculations, humidity around the plants was maintained using V5100NS humidifiers (Vicks Ultrasonic Humidifiers, Hudson, NY, USA) from 24 h before inoculation to 48 h after inoculation and by covering the seedlings with clear plastic. Four to six weeks after transplanting, the seedlings were sprayed with the bacterial suspension until foliar runoff using a hand sprayer around sunset. Sterile water was used for mock inoculation. Leaf tissue samples after inoculation were collected in liquid nitrogen and stored at −80 °C until further processed.

Greenhouse plants were scored for foliar symptoms on the most severely infected leaves using the Horsfall–Barratt scale, where 0% = 1, 1–3% = 2, 3–6% = 3, 6–12% = 4, 12–25% = 5, 25–50% = 6, 50–75% = 7, 75–87% = 8, 87–94% = 9, 94–97% = 10, 97–100% = 11 and 100% dead tissue = 12 [51]. However, the data is reported in percentage.

Area under the disease progress curve was calculated based on weekly disease severity assessments. AUDPC is a quantitative summary of disease severity over time and compares average disease severity between pairs of adjacent time points [52]. The AUDPC is calculated as follows:

$$AUDPC=\overset{n-1}{\underset{i=1}{\sum }}\frac{y_{i}+y_{i+1}}{2}\times \left(t_{i+1}-t_{i}\right)$$

where *y_i_* is the assessment of the disease at the *i*th observation, *t_i_* is the time at the *i*th observation, and n is the total number of observations.

### 4.3. RNA Extraction and RNA-Seq Library Construction

Leaves from three tomato genotypes were collected in liquid nitrogen as three replicates at 48 h after inoculation (hai). Frozen samples were stored in a −80 °C freezer. Before RNA extraction, frozen leaves were ground into fine powder by using pestle and mortar in liquid nitrogen. About 100 mg ground tissue sample was transferred into a 1.5 mL tube for the extraction of total RNA using the Qiagen Plant RNeasy mini kit (Qiagen, Hilden, Germany). The RNA quality and quantity were evaluated by using Nanodrop (Fisher Scientific, Waltham, MA, USA) and MOPs gel electrophoresis [53]. Total RNA samples were used for RNA-Seq library construction using NEBNext^®^ Ultra^TM^ Directional RNA Library Prep Kit for Illumina (New England BioLabs, Ipswich, MA, USA). We followed the protocol for the normal insertion size.

### 4.4. RNA-Seq Deep Sequencing, Data Processing, Mapping, and Differential Gene Expression Analysis

RNA-Seq data was generated on Illumina HiSeq2500 instruments in 150 bp read mode at Genomic Sciences Laboratory, North Carolina State University, Raleigh, NC, USA and reads were provided in FASTq format. All reads were quality checked using FastQC [54], and trimmed to eliminate poor quality bases (Q30) using fastq-mcf function.

Reads were mapped against tomato Heinz 1706 genome assembly SL3.0 using Hisat2 version 2.1.0 [55]. Reads mapped to gene assembly were manipulated using Samtools [56] for sorting/indexing, and the raw count of reads mapped to the annotated gene model (ITAG3.2 version) was extracted using Bedtools version 2.25.0 [57]. Information on raw counts of mapped RNA-Seq reads to annotated gene models were analyzed using R package EdgeR [22], including exploration of RNA-Seq libraries relationship using the plotMDS function, gene expression normalization followed TMM algorithm [58], and differentially expressed gene (DEG) identification followed classical approach. Criteria of DEG is gene expression fold change >2.0 times between the compared group of samples, and statistics level in the form of false discovery rate (FDR) <0.05.

### 4.5. Gene Functional and Pathway Analysis

Biological function evaluation for DEGs was conducted using the online GO analysis toolkit AgriGO2.0 [59] following an option of using ITAG3.2 version transcript ID and suggested ITAG3.2 background.

MapMan software (version 3.6.0RC1) was used to evaluate the DEGs’ function in the different pathways [60] using a list of DEGs with fold change in log2 between control and inoculated samples for each tomato line as input data. Gene annotation in the pathway analysis was prepared via Mercator online software within the PlabiPD website (http://www.plabipd.de) based on the ITAG3.2 protein sequence and followed the default annotation parameter.

### 4.6. SNP/INDEL Identification

Sequence variations were extracted using the mpileup function of Samtools package [56] from the mapping result file in the format as BAM. For even comparison, these BAM files were the result of mapping the same amount of clean RNA-Seq reads (20 million) from two control RNA-Seq libraries for each tomato line. To get high-quality SNP/INDELs, data in raw variant calling format (VCF, version 4.0) files were filtered for minimum depth (DP) 10 and SNP/INDELs quality (Q) over 30 [18]. To screen SNP/INDELs specific to individual tomato lines, loci with homozygous SNP/INDEL against Heinz 1706, i.e., in the form of 1/1 in genotype (GT) section of VCF output file), were selected.

Genes with SNP/INDELs were screened using vlookup function in Excel environment, and location information of genes with SNP/INDELs on the chromosome was converted into CSV format and used as input for R QTL package [61] to generate a genetic map for visualization. Online software Venny2.1 online [62] and BioVenn [63] were used to generate Venn diagrams or extract overlapping information for DEGs, SNP/INDELs.

## 5. Conclusions

We selected three distinct tomato lines based on their different response to BS. We performed RNA-Seq analysis for these lines to investigate DEGs induced two days after inoculation with BS race T4. Comparing functional involvement in various processes and past studies, we identified unique differentially expressed genes in resistance accession PI 270443, such as upregulated PR-protein genes (solyc09g092300, solyc08g081790, and solyc09g092310) specific to this study. On the other hand, a disease-associated R gene (solyc12g097000) was found downregulated in susceptible line NC 714. In addition to these differentially expressed genes, we used transcriptome-based RNA-Seq analysis to call SNP/INDELs from expressed genes between the three different tomato lines and found that most of the molecular markers from resistant tomato lines PI 270443 and NC 1CELBR were on chr11. Several biotic stress-associated genes, including PR-protein genes, were identified with sequence variation in addition to known *Rx4* resistance genes. All these findings are a valuable resource for tomato breeding aiming to develop plants resistant to BS race T4.

## Figures and Tables

**Table 1:** Bacterial spot disease development in the tomato breeding lines. Tomato plants were inoculated 15 days after planting (dap). While the first symptom was observed on different days in different lines, we started scoring the lines for bacterial spot (BS) six days after inoculation (dai) of the plants and scored in every two days interval. Data presented in percentage. Last column has the area under disease progress curve (AUDPC) data, which can be used as a single indicator in disease screening.

Genotype	First Symptom dai	6 dai (21 dap)	9 dai (24 dap)	11 dai (26 dap)	13 dai (28 dap)	15 dai (30 dap)	17 dai (32 dap)	19 dai (34 dap)	AUDPC
PI 270443	7.0	1.0	1.0	4.5	10.0	22.5	20.0	17.5	135.5
PI 114490	6.0	1.0	1.0	12.5	20.0	22.5	35.0	37.5	221.5
CLN-2413A	7.0	1.0	3.0	8.0	22.5	27.5	32.5	32.5	222.5
LA2093	5.5	1.0	1.0	4.5	20.0	32.5	35.0	35.0	223.0
LA4277	6.0	1.0	1.5	10.0	17.5	42.5	42.5	37.5	267.8
Fla 7060_Xv4	6.0	1.0	3.0	10.0	15.0	35.0	50.0	50.0	279.0
NC 25P	6.0	1.0	3.0	17.5	32.5	37.5	45.0	47.5	321.5
NCEBR8	6.5	1.0	3.0	17.5	25.0	45.0	50.0	42.5	326.5
CLN-2418A	6.0	1.0	10.5	20.5	35.0	40.0	45.0	42.5	351.3
NC161L	6.5	2.0	8.0	25.5	30.0	40.0	47.5	47.5	356.5
NCEBR6	7.0	1.0	3.0	12.5	42.5	45.0	50.0	50.0	359.0
Money maker	6.0	1.0	9.5	27.5	35.0	45.0	52.5	52.5	397.8
LA2653	5.5	1.5	10.0	17.5	50.0	45.0	50.0	47.5	399.8
NC123S	6.5	1.0	15.5	22.5	32.5	52.5	47.5	50.0	400.3
VF36	6.5	10.0	5.5	25.5	35.0	47.5	52.5	52.5	402.3
Pto-S	5.5	1.0	6.0	17.5	40.0	60.0	57.5	55.0	421.5
Hawaii 7998	6.0	1.5	15.5	30.5	45.0	47.5	50.0	35.0	422.0
CLN-1466EA	6.0	1.0	20.5	32.0	42.5	42.5	47.5	45.0	426.8
NC 1CELBR	6.0	1.5	16.0	32.5	47.5	37.5	50.0	55.0	432.3
NC 30P	7.0	1.0	10.5	20.5	40.0	57.5	57.5	55.0	433.8
NC 1CS	6.5	1.0	10.5	21.0	42.5	55.0	57.5	55.0	434.8
NC 22L-1(2008)	6.5	5.0	10.5	25.0	52.5	57.5	60.0	55.0	478.8
HI7997	6.0	1.0	11.0	22.5	50.0	60.0	60.0	67.5	481.5
Pto-R	6.0	8.0	20.0	41.0	47.5	57.5	60.0	65.0	539.0
Hawaii 7981	6.0	5.5	17.0	50.0	60.0	70.0	65.0	67.5	608.3
NCEBR7	6.0	1.0	9.5	25.0	50.0	85.0	90.0	95.0	620.3
NC 6Grape	6.0	15.0	20.5	40.0	65.0	77.5	77.5	75.0	668.8
NC 714	5.5	6.0	15.0	40.0	65.0	85.0	87.5	85.0	686.5
NC 5Grape	5.5	8.0	25.0	55.0	67.5	72.5	80.0	80.0	704.5
Heinz 1706	6.0	3.5	25.0	55.0	67.5	87.5	92.5	90.0	762.8

**Table 2:** Summary of RNA-Seq library information.

Sample ID	No. of Raw Reads	No. of Clean Reads	No. of Reads Mapped to the Genome	No. of Reads Mapped to Genome after Filtering
PI270443—CK1	17,401,320	17,366,776	15,679,410	14,493,893
PI270443—CK2	16,652,433	16,627,277	14,877,660	14,493,893
NCCELBR1—CK1	22,569,408	22,539,261	20,742,997	20,134,965
NCCELBR1—CK2	15,898,554	15,872,147	13,799,117	13,418,896
NC 714—CK1	16,716,909	16,672,953	15,014,160	14,652,561
NC 714—CK2	8,045,470	8,028,488	6,783,492	6,615,839
PI270443—IN1	15,187,278	15,150,128	12,830,082	12,458,547
PI270443—IN2	10,417,144	10,390,478	8,475,406	8,204,475
NCCELBR1—IN1	26,100,659	26,015,053	24,082,176	23,340,952
NCCELBR1—IN2	37,792,194	37,721,275	35,177,779	34,073,612
NC 714—IN1	57,979,118	57,873,646	54,422,298	52,813,125
NC 714—IN2	22,812,966	22,762,930	19,933,275	19,364,162

**Table 3:** DEGs in the biotic stress pathway associated with resistance and susceptibility. The upper part of the table lists all upregulated biotic stress pathway DEGs in three lines with expression change (log2) between inoculated and control samples, and the lower part of the table list all downregulated biotic stress pathway DEGs in the susceptible line NC 714. Expression change in the blue and red background is for upregulated and downregulated DEGs, respectively. The intensity of the two colors indicates the level of expression of genes changes in both directions.

**Upregulated** **Gene Loci**	**PI ** **270443**	**NC 1CELBR**	**NC 714**	**Gene Annotation Bin Defined in MapMan Analysis**
solyc10g085870	1.1	1.6	2.3	secondary metabolism.flavonoids.flavonols
solyc05g008120	1.4	1.7	1.7	stress.abiotic.heat
solyc12g006380	1.3	1.4		hormone metabolism.ethylene.synthesis-degradation
solyc04g078140	2.9	1.5		redox.ascorbate and glutathione
solyc02g081340	2.5	1.1		misc.glutathione S transferases
solyc07g056440	1.0	1.2		misc.glutathione S transferases
solyc01g058720	1.9	1.5		signalling.calcium
solyc09g082660	3.6	1.6		secondary metabolism.phenylpropanoids.lignin biosynthesis.CCoAOMT
solyc09g059170	1.1	1.2		secondary metabolism.flavonoids.anthocyanins
solyc08g080040	2.8	1.7		secondary metabolism.flavonoids.anthocyanins.leucocyanidin dioxygenase
solyc05g053550	2.1	1.3		secondary metabolism.flavonoids.chalcones.naringenin-chalcone synthase
solyc09g091510	1.9	1.0		secondary metabolism.flavonoids.chalcones.naringenin-chalcone synthase
solyc02g085020	4.1	1.4		secondary metabolism.flavonoids.dihydroflavonols.dihydroflavonol 4-reductase
solyc02g083860	1.2	1.0		secondary metabolism.flavonoids.dihydroflavonols.flavanone 3-hydroxylase
solyc11g066580	3.0	1.7		secondary metabolism.flavonoids.dihydroflavonols.flavonoid 3-monooxygenase
solyc10g083440	1.3	1.0		secondary metabolism.flavonoids.flavonols.flavonol-3-O-rhamnosyltransferase
solyc12g098590	4.0	1.5		hormone metabolism.auxin.synthesis-degradation
solyc08g062340	3.4	2.1		stress.abiotic.heat
solyc08g074990	1.3		1.9	cell wall.pectin*esterases.acetyl esterase
solyc09g008990	1.4			cell wall.cellulose synthesis
solyc09g009010	1.2			cell wall.cellulose synthesis
solyc10g083670	1.1			cell wall.cellulose synthesis
solyc02g080160	1.1			cell wall.modification
solyc11g065970	1.3			secondary metabolism.simple phenols
solyc12g089050	1.7			secondary metabolism.wax
solyc06g082420	1.7			misc.glutathione S transferases
solyc02g070870	1.5			protein.targeting.secretory pathway.unspecified
solyc09g010620	1.7			protein.postranslational modification
solyc05g051580	2.0			signalling.light
solyc03g115930	2.2			signalling.calcium
solyc06g084450	1.3			signalling.G-proteins
solyc11g013740	1.2			signalling.G-proteins
solyc09g007420	1.6			cell wall.hemicellulose synthesis.glucuronoxylan
solyc06g075220	1.1			cell wall.cell wall proteins.AGPs.AGP
solyc04g080620	3.1			cell wall.degradation.mannan-xylose-arabinose-fucose
solyc11g066670	1.2			secondary metabolism.phenylpropanoids.lignin biosynthesis
solyc06g083445	2.0			misc.O-methyl transferases
solyc06g083450	1.7			misc.O-methyl transferases
solyc10g086270	1.5			secondary metabolism.flavonoids.anthocyanins
solyc05g010320	1.2			secondary metabolism.flavonoids.chalcones
solyc02g090890	1.1			hormone metabolism.abscisic acid.synthesis-degradation.synthesis.zeaxanthin epoxidase
solyc08g079150	1.4			hormone metabolism.auxin.induced-regulated-responsive-activated
solyc01g110570	1.3			hormone metabolism.auxin.induced-regulated-responsive-activated
solyc11g006300	1.3			hormone metabolism.brassinosteroid.synthesis-degradation.BRs.DET2
solyc01g104740	1.5			hormone metabolism.ethylene.induced-regulated-responsive-activated
solyc09g092300	1.6			stress.biotic
solyc09g092310	1.4			stress.biotic
solyc08g081790	1.6			stress.biotic.PR-proteins
solyc06g072330	1.7			stress.abiotic.heat
solyc03g123540	1.6			stress.abiotic.heat
solyc08g078695	1.5			stress.abiotic.heat
solyc11g066100	1.2			stress.abiotic.heat
solyc05g005865	1.3			stress.abiotic.unspecified
solyc06g009710	1.5			RNA.regulation of transcription.MYB domain transcription factor family
solyc10g076370	2.4			RNA.regulation of transcription.AP2/EREBP, APETALA2/Ethylene-responsive element binding protein family
solyc03g120840	1.5			RNA.regulation of transcription.AP2/EREBP, APETALA2/Ethylene-responsive element binding protein family
solyc05g052410	1.3			RNA.regulation of transcription.AP2/EREBP, APETALA2/Ethylene-responsive element binding protein family
solyc08g082210	1.1			RNA.regulation of transcription.AP2/EREBP, APETALA2/Ethylene-responsive element binding protein family
solyc09g059630	1.2			protein.degradation.ubiquitin
solyc12g010500	1.8			protein.degradation.ubiquitin.E3.RING
solyc04g007500	1.4			protein.degradation.ubiquitin.E3.RING
solyc10g085660	1.1			protein.degradation.ubiquitin.E3.SCF.FBOX
solyc01g104230	1.0			protein.degradation.ubiquitin.E3.SCF.FBOX
solyc06g071830	1.4			protein.degradation.ubiquitin.E3.BTB/POZ Cullin3.BTB/POZ
solyc06g072016	1.3			protein.degradation.AAA type
solyc02g079990	4.1			signalling.receptor kinases.DUF 26
solyc11g012020	1.0			signalling.calcium
**Downregulated Gene loci**	**PI ** **270443**	**NC 1CELBR**	**NC 714**	**Gene annotation (Bin defined in MapMan)**
solyc01g105070	−2.6	−2.0	−3.1	misc.peroxidases
solyc10g078220	−4.1	−2.5	−3.4	secondary metabolism.phenylpropanoids.lignin biosynthesis.C3H
solyc12g042480	−1.5	−2.0	−1.6	secondary metabolism.phenylpropanoids.lignin biosynthesis.F5H
solyc05g052040	−2.4	−1.6	−2.2	hormone metabolism.ethylene.signal transduction
solyc01g106620	−3.7	−2.3	−2.5	stress.biotic
solyc04g054690	−2.5	−-2.0	−2.7	redox.ascorbate and glutathione.ascorbate
solyc01g059965	−4.4	−2.3	−3.2	misc.beta 1,3 glucan hydrolases.glucan endo-1,3-beta-glucosidase
solyc01g059980	−4.4	−2.3	−3.2	misc.beta 1,3 glucan hydrolases.glucan endo-1,3-beta-glucosidase
solyc01g060020	−4.4	−2.3	−3.2	misc.beta 1,3 glucan hydrolases.glucan endo-1,3-beta-glucosidase
solyc02g036370	−1.3	−1.5	−2.8	RNA.regulation of transcription.MYB-related transcription factor family
solyc05g009790	−1.6	−1.7	−2.0	RNA.regulation of transcription.AP2/EREBP, APETALA2/Ethylene-responsive element binding protein family
solyc03g095770	−2.8	−1.1	−1.9	RNA.regulation of transcription.WRKY domain transcription factor family
solyc09g014990	−1.8	−2.7	−4.2	RNA.regulation of transcription.WRKY domain transcription factor family
solyc03g083470	−1.7	−2.2	−2.3	signalling.receptor kinases.wheat LRK10 like
solyc05g009010	−1.1	−1.1	−1.6	signalling.receptor kinases.wheat LRK10 like
solyc01g006300		−1.0	−1.5	misc.peroxidases
solyc03g113950		−1.3	−1.2	signalling.calcium
solyc05g007710		−2.0	−3.4	RNA.regulation of transcription.MYB domain transcription factor family
solyc01g095630		−1.1	−1.4	RNA.regulation of transcription.WRKY domain transcription factor family
solyc08g008280		−1.3	−1.6	RNA.regulation of transcription.WRKY domain transcription factor family
solyc12g055710		−3.3	−4.0	protein.degradation.ubiquitin.E3.RING
solyc02g076980		−2.5	−2.5	protein.degradation.cysteine protease
solyc02g080040		−2.5	−1.7	signalling.receptor kinases.DUF 26
solyc08g080670	−2.5		−2.5	stress.abiotic
solyc11g018775	−2.5		−3.2	misc.glutathione S transferases
solyc11g018777	−2.3		−3.2	misc.glutathione S transferases
solyc11g018800	−2.9		−2.7	misc.glutathione S transferases
solyc11g018805	−2.3		−3.2	misc.glutathione S transferases
solyc04g048900	−1.7		−1.6	signalling.calcium
solyc10g006700	−1.2		−1.2	signalling.calcium
solyc01g095580	−1.0		−1.1	hormone metabolism.auxin.induced-regulated-responsive-activated
solyc08g008087	−1.4		−1.4	hormone metabolism.ethylene.synthesis-degradation.1-aminocyclopropane-1-carboxylate synthase
solyc03g093560	−1.7		−1.9	hormone metabolism.ethylene.signal transduction
solyc05g051200	−2.9		−1.9	hormone metabolism.ethylene.signal transduction
solyc05g052050	−1.5		−1.4	hormone metabolism.ethylene.signal transduction
solyc08g078190	−1.0		−2.1	hormone metabolism.ethylene.signal transduction
solyc07g008590	−1.3		−1.4	stress.biotic.PR-proteins
solyc07g008620	−2.5		−2.3	stress.biotic.PR-proteins
solyc03g098730	−.11		−2.9	stress.biotic.PR-proteins.proteinase inhibitors.trypsin inhibitor
solyc07g055710	−1.8		−2.7	stress.abiotic.heat
solyc08g023660	−1.3		−1.2	stress.biotic
solyc09g090130	−1.7		−1.6	RNA.regulation of transcription.MYB domain transcription factor family
solyc06g068460	−2.1		−1.4	RNA.regulation of transcription.WRKY domain transcription factor family
solyc09g015770	−2.2		−1.8	RNA.regulation of transcription.WRKY domain transcription factor family
solyc08g079860	−1.0		−3.1	protein.degradation.subtilases
solyc08g079870	−2.3		−3.3	protein.degradation.subtilases
solyc01g066430	−1.1		−1.8	protein.degradation.ubiquitin.E3.RING
solyc11g068710	−1.2		−1.7	protein.degradation.ubiquitin.E3.SCF.FBOX
solyc08g068860	−1.7		−1.2	RNA.regulation of transcription.unclassified
solyc03g033790	−3.0		−2.6	protein.degradation.AAA type
solyc04g074000	−1.9		−2.6	signalling.receptor kinases.leucine rich repeat XII
solyc04g074050	−1.2		−1.9	signalling.receptor kinases.leucine rich repeat XII
solyc02g014030	−2.7		−2.0	signalling.receptor kinases.Catharanthus roseus-like RLK1
solyc09g011330	−1.3		−1.4	misc.myrosinases-lectin-jacalin
solyc01g067020	−1.9		−2.0	signalling.receptor kinases.leucine rich repeat III
solyc11g066270			−1.3	cell wall.modification
solyc08g080640			-3.2	stress.abiotic
solyc08g080650			−1.6	stress.abiotic
solyc12g019740			−1.0	redox.thioredoxin
solyc04g009860			−1.4	hormone metabolism.ethylene.synthesis-degradation
solyc07g008240			−1.1	redox.heme
solyc07g053550			−1.6	redox.glutaredoxins
solyc04g071890			−1.2	misc.peroxidases
solyc09g011560			−1.9	misc.glutathione S transferases
solyc09g011590			−1.5	misc.glutathione S transferases
solyc09g011630			−1.3	misc.glutathione S transferases
solyc00g187050			−2.5	protein.degradation
solyc03g025670			−2.0	signalling.in sugar and nutrient physiology
solyc03g118810			−1.1	signalling.calcium
solyc03g119250			−1.7	signalling.calcium
solyc10g006660			−1.6	signalling.calcium
solyc10g079420			−1.1	signalling.calcium
solyc06g005170			−1.2	signalling.MAP kinases
solyc12g017240			−1.2	cell wall.degradation.mannan-xylose-arabinose-fucose
solyc04g014400			−1.4	cell wall.degradation.pectate lyases and polygalacturonases
solyc07g052230			−1.0	cell wall.pectin*esterases.PME
solyc02g093230			−1.4	secondary metabolism.phenylpropanoids.lignin biosynthesis.CCoAOMT
solyc02g093250			−2.4	secondary metabolism.phenylpropanoids.lignin biosynthesis.CCoAOMT
solyc02g093270			−1.1	secondary metabolism.phenylpropanoids.lignin biosynthesis.CCoAOMT
solyc04g078290			−1.4	secondary metabolism.sulfur-containing.glucosinolates.synthesis.indole.cytochrome P450 monooxygenase
solyc03g080190			−1.7	secondary metabolism.flavonoids.dihydroflavonols
solyc07g049530			−2.5	hormone metabolism.ethylene.synthesis-degradation.1-aminocyclopropane-1-carboxylate oxidase
solyc03g093540			−1.3	hormone metabolism.ethylene.signal transduction
solyc03g093550			−1.2	hormone metabolism.ethylene.signal transduction
solyc04g014530			−1.3	hormone metabolism.ethylene.signal transduction
solyc07g053740			−1.0	hormone metabolism.ethylene.signal transduction
solyc10g009110			−1.4	hormone metabolism.ethylene.signal transduction
solyc01g006540			−1.3	hormone metabolism.jasmonate.synthesis-degradation.lipoxygenase
solyc12g097000			−1.7	stress.biotic
solyc10g076500			−1.7	stress.biotic
solyc02g090380			−-1.1	stress.biotic
solyc04g007320			−1.5	stress.biotic.PR-proteins
solyc06g068500			−1.2	stress.abiotic.heat
solyc08g008370			−1.1	stress.abiotic.touch/wounding
solyc01g008620			−3.4	misc.beta 1,3 glucan hydrolases.glucan endo-1,3-beta-glucosidase
solyc09g008250			−1.7	RNA.regulation of transcription.MYB domain transcription factor family
solyc03g116890			−3.1	RNA.regulation of transcription.WRKY domain transcription factor family
solyc04g051690			−3.6	RNA.regulation of transcription.WRKY domain transcription factor family
solyc06g066370			−1.5	RNA.regulation of transcription.WRKY domain transcription factor family
solyc08g067340			−3.9	RNA.regulation of transcription.WRKY domain transcription factor family
solyc08g082110			−2.7	RNA.regulation of transcription.WRKY domain transcription factor family
solyc01g087810			−1.2	protein.degradation.subtilases
solyc08g079900			−4.2	protein.degradation.subtilases
solyc06g074770			−1.4	protein.degradation.ubiquitin
solyc11g005640			−1.3	protein.aa activation
solyc01g079530			−1.2	protein.degradation.ubiquitin.E3.RING
solyc03g034020			−1.0	protein.degradation.ubiquitin.E3.RING
solyc11g010330			−1.4	protein.degradation.ubiquitin.E3.RING
solyc05g005150			−2.1	protein.degradation.ubiquitin.E3.SCF.FBOX
solyc03g111710			−1.1	protein.degradation.ubiquitin.E3.BTB/POZ Cullin3.BTB/POZ
solyc02g077040			−2.9	protein.degradation.cysteine protease
solyc11g066250			−1.1	protein.degradation.serine protease
solyc02g087540			−2.8	protein.degradation.AAA type
solyc10g007280			−2.3	protein.degradation.AAA type
solyc04g076990			−1.1	signalling.receptor kinases.leucine rich repeat XI
solyc04g074020			−2.1	signalling.receptor kinases.leucine rich repeat XII
solyc04g074030			−2.7	signalling.receptor kinases.leucine rich repeat XII
solyc09g072810			−1.4	signalling.receptor kinases.leucine rich repeat XII
solyc02g080010			−1.9	signalling.receptor kinases.DUF 26
solyc05g009000			−1.5	signalling.receptor kinases.wheat LRK10 like
solyc03g078360			−2.1	protein.postranslational modification
solyc11g005630			−1.7	misc.myrosinases-lectin-jacalin
solyc10g076550			−1.5	signalling.receptor kinases.wall associated kinase
solyc11g020230			−1.2	signalling.receptor kinases.crinkly like
solyc08g016210			−1.9	stress.biotic

**Table 4:** Effector-triggered immunity (ETI) and pattern-triggered immunity (PTI)-associated genes among BS-induced upregulated DEGs. DEG expression change listed as (log2) between inoculated and control samples underlines PI270443, NC 1CELBR, and NC714. The intensity of the blue background represents the degree of upregulation.

Loci ID	Layer	PI 270443	NC 1CELBR	NC 714	Function Annotation (ITAG3.2)
Solyc02g079990	ETI	4.09			Cysteine-rich recETIor-kinase-like protein
Solyc03g115930	ETI	2.17			Calcium-binding EF-hand family protein
Solyc07g063170	ETI	2.01			Sodium/calcium exchanger family protein
Solyc01g107400	ETI	1.98			IAA-amido synthetase
Solyc01g109120	ETI	1.90			Transducin/WD40 repeat-like superfamily protein
Solyc11g069700	ETI	1.83	1.41		Elongation factor 1-alpha
Solyc10g080370	ETI	1.60			LOW QUALITY:Transmembrane protein, putative
Solyc01g104740	ETI	1.52	1.63		Multiprotein-bridging factor, putative
Solyc09g082710	ETI	1.52			Histone H2A
Solyc04g082380	ETI	1.28			BnaA03g07530D protein
Solyc12g006380	ETI	1.27	1.43		2-oxoglutarate-dependent dioxygenase
Solyc02g092110	ETI	1.25			Phytosulfokines 3 family protein
Solyc02g083860	ETI	1.22	1.02		flavanone 3-dioxygenase
Solyc06g075800	ETI	1.17		1.03	Histone H2B
Solyc07g065410	ETI	1.15			LOW QUALITY:Melanin-concentrating hormone recETIor 1
Solyc08g082210	ETI	1.14			AP2/EREBP transcription factor
Solyc03g116170	ETI	1.14			Nucleosome assembly protein family
Solyc03g096670	ETI	1.14			Protein phosphatase 2C
Solyc02g080150	ETI	1.14			CAI-1 autoinducer sensor kinase/phosphatase cqsS isoform 1
Solyc06g072430	ETI	1.11	1.22		BAG family molecular chaperone regulator 5
Solyc10g007010	ETI	1.11			Cytochrome c oxidase copper chaperone, putative
Solyc11g066840	ETI	1.11			Histone deacetylase-like protein-like
Solyc03g120390	ETI	1.10			Auxin responsive protein IPR003311 AUX_IAA protein
Solyc10g085870	ETI	1.09	1.56	2.30	Glycosyltransferase
Solyc12g042650	ETI	1.08			40S ribosomal protein S12
Solyc10g006560	ETI	1.06			Histone H2A
Solyc10g018810	ETI	1.05			60S ribosomal protein L7A-like protein
Solyc01g091840	ETI	1.05			UDP-galactose transporter
Solyc12g005270	ETI	1.02			Histone H2A
Solyc03g007770	ETI	1.01			S-type anion channel
Solyc10g085880	ETI		2.73		Glycosyltransferase
Solyc12g062520	ETI		2.64		AP-2 complex subunit mu
Solyc07g056430	ETI		1.15		Glutathione S-transferase-like protein
Solyc09g092500	ETI		1.14	1.09	Glycosyltransferase
Solyc07g056480	ETI		1.03		glutathione S-transferase/peroxidase
Solyc09g092490	ETI		1.03		Glycosyltransferase
Solyc11g069960	ETI			1.82	RLK-1
Solyc10g086410	ETI			1.42	LEHSC270 hsc-2heat shock protein cognate 70
Solyc06g076020	ETI			1.41	heat shock protein 70 kD
Solyc09g092520	ETI			1.39	xyloglucan endotransglycosylase
Solyc11g066060	ETI			1.39	heat shock protein 70
Solyc09g005120	ETI			1.21	DnaJ domain-containing protein
Solyc05g007150	ETI			1.06	UDP-galactose transporter, putative
Solyc08g065850	ETI			1.00	Arabinogalactan pETIide 14
Solyc08g062340	ETI-PTI	3.40	2.12		Heat-shock protein, putative
Solyc01g058720	ETI-PTI	1.93	1.50		Calcium-binding EF-hand
Solyc06g072330	ETI-PTI	1.70			DNAJ protein, putative, expressed
Solyc01g086670	ETI-PTI	1.57			LOW QUALITY:Expressed protein-RZ53
Solyc02g089660	ETI-PTI	1.27			Titin
Solyc11g008530	ETI-PTI	1.11			Dicer-like 2d
Solyc08g075540	ETI-PTI	1.08			alternative oxidase 1au
Solyc01g107780	ETI-PTI		3.08		Glycosyltransferase
Solyc06g008620	ETI-PTI		1.74		LOW QUALITY:tolB protein-like protein
Solyc01g109090	ETI-PTI		1.38		LOW QUALITY:mRNA, clone: RTFL01-34-C05
Solyc12g042600	ETI-PTI		1.33		Glycosyltransferase
Solyc07g008440	PTI	1.03			Purine permease-like protein

**Table 5:** Distribution of single nucleotide polymorphisms (SNP)/insertion–deletions (INDELs) on different chromosomes of tomato.

	SNP/INDELs Loci (Gene with SNP/INDELs in Parentheses)			
Chr	Total	PI 270443	NC 1CELBR	NC 714
1	1850 (728)	781 (274)	734 (248)	1219 (481)
2	1285 (531)	437 (167)	563 (191)	875 (397)
3	2162 (720)	595 (230)	739 (247)	1575 (584)
4	3306 (825)	1143 (298)	2061 (488)	1826 (556)
5	1266 (455)	633 (168)	544 (161)	958 (376)
6	1149 (432	414 (125)	378 (110)	949 (379)
7	1242 (366)	447 (146)	398 (143)	937(309)
8	1359 (466)	517 (139)	559 (152)	1036 (400)
9	2210 (535)	675 (200)	1522 (345)	1264 (397)
10	1869 (473)	825 (242)	1244 (289)	904 (273)
11	3835 (716)	2893 (544)	2894 (545)	1699 (341)
12	1223 (377)	944 (311)	938 (301)	488 (152)
Total	23,253 (6676)	10,666 (2865)	12,942 (3239)	14,063 (4667)

**Table 6:** Biotic stress pathway DEGs or genes with SNP/INDELs on chr11 in position (POS) listed as Million base (Mb). In the section of SNP/INDELs, “S”, “I”, and “D” represent sequence variation in the homo form of SNP, INSERT, and DELETION alternative form against the reference genome of Heinz 1706. The DEG fold changes are log_2_ of gene expression change induced by BS inoculation (IN/CK). Gene functions annotation bin were annotated by MapMan package in this study. DEG fold change background color intensity corresponds to the level of change.

Loci_ID	POS Mb	SNP/INDEL			DEG Fold Change			Function Annotation Bin in MapMan for Biotic Stress Pathway Genes
		PI 270443	NC 1CELBR	NC 714	PI 270443	NC 1CELBR	NC 714	
solyc11g005010	0.0			S				signalling.G-proteins
solyc11g005060	0.1			S				hormone metabolism.auxin.induced-regulated-responsive-activated
solyc11g005130	0.1			S				stress.abiotic.touch/wounding
solyc11g005150	0.3			I				cell wall.cell wall proteins.LRR
solyc11g005630	0.5			S				signalling.receptor kinases.S-locus glycoprotein like
solyc11g005640	0.5						−1.3	protein.degradation.ubiquitin
solyc11g005910	0.7			S				signalling.phosphinositides.phosphatidylinositol 4-kinase
solyc11g006180	0.9			S				hormone metabolism.ethylene.signal transduction
solyc11g006300	1.0				1.3			hormone metabolism.brassinosteroid.synthesis-degradation.BRs.DET2
solyc11g006590	1.2			S				secondary metabolism.sulfur-containing.glucosinolates.synthesis.shared. Phenylacetaldoxime monooxygenase
solyc11g008250	2.5			S				protein.degradation
solyc11g008260	2.5			S				protein.degradation.cysteine protease
solyc11g008280	2.6			S				protein.degradation
solyc11g008450	2.7			S				redox.thioredoxin
solyc11g008850	3.0			S				protein.degradation.serine protease
solyc11g008960	3.1			S				signalling.receptor kinases.leucine rich repeat II
solyc11g010310	3.3			S				protein.degradation.ubiquitin.E3.RING
solyc11g010330	3.4	I	I	I			−1.4	protein.degradation.ubiquitin.E3.RING
solyc11g010470	3.5			S				signalling.14-3-3 proteins
solyc11g010480	3.5			SI				protein.degradation
solyc11g010600	3.7			S				hormone metabolism.auxin.induced-regulated-responsive-activated
solyc11g010650	3.7	DS	DS	DS				secondary metabolism.phenylpropanoids
solyc11g010740	3.8		SD	A				secondary metabolism.flavonoids.anthocyanins
solyc11g010760	3.8		S	A				secondary metabolism.flavonoids.anthocyanins
solyc11g010790	3.8	S		S				secondary metabolism.flavonoids.anthocyanins
solyc11g010810	3.8			S				secondary metabolism.flavonoids.anthocyanins
solyc11g010850	3.9			S				secondary metabolism.isoprenoids.non-mevalonate pathway.DXS
solyc11g010940	4.0			S				RNA.regulation of transcription.C2C2(Zn) DOF zinc finger family
solyc11g010960	4.0			S				secondary metabolism.phenylpropanoids.lignin biosynthesis.CAD
solyc11g011000	4.0			S				signalling.receptor kinases.misc
solyc11g011020	4.0							signalling.receptor kinases.leucine rich repeat III
solyc11g011050	4.1			A				RNA.regulation of transcription.MYB domain transcription factor family
solyc11g011060	4.2			SD				stress.biotic.PR-proteins
solyc11g011080	4.2			S				stress.biotic.PR-proteins
solyc11g011120	4.2			SI				signalling.calcium
solyc11g011200	4.3			SI				stress.abiotic
solyc11g011250	4.3			S				redox.ascorbate and glutathione.ascorbate
solyc11g011260	4.3			S				signalling.in sugar and nutrient physiology
solyc11g011340	4.4			S				secondary metabolism.phenylpropanoids.lignin biosynthesis.CAD
solyc11g011440	4.5			S	−1.2			protein.degradation.aspartate protease
solyc11g011508	4.6			S				protein.degradation.ubiquitin.E3.SCF.FBOX
solyc11g011540	4.6			S				protein.degradation.ubiquitin.E3.SCF.FBOX
solyc11g011546	4.6			S				protein.degradation.ubiquitin.E3.SCF.FBOX
solyc11g011548	4.6			S				protein.degradation.ubiquitin.E3.SCF.FBOX
solyc11g011630	4.7			S				hormone metabolism.auxin.induced-regulated-responsive-activated
solyc11g011640	4.7		S	S				hormone metabolism.auxin.induced-regulated-responsive-activated
solyc11g011670	4.7			S				hormone metabolism.auxin.induced-regulated-responsive-activated
solyc11g011700	4.7	S	S	S				hormone metabolism.auxin.induced-regulated-responsive-activated
solyc11g011850	4.8	S	S	?				cell wall.hemicellulose synthesis.glucuronoxylan
solyc11g011880	4.8	S	S	S				signalling.receptor kinases.DUF 26
solyc11g012020	5.0	S	S		1.0			signalling.calcium
solyc11g012040	5.0	S	S					protein.degradation.ubiquitin
solyc11g012410	5.2	SD	SD					redox.ascorbate and glutathione.ascorbate.L-galactose-1-phosphate phosphatase
solyc11g012460	5.3	IS	IS					signalling.G-proteins
solyc11g012510	5.3	S	S					signalling.light
solyc11g012550	5.4	S	S					protein.degradation.ubiquitin.E3.SCF.FBOX
solyc11g012580	5.4	S	S					misc.beta 1,3 glucan hydrolases
solyc11g012710	5.5	S	S					signalling.in sugar and nutrient physiology
solyc11g013010	5.9	S	S					signalling.phosphinositides.phosphatidylinositol-4-phosphate 5-kinase
solyc11g013170	6.0	S	S					secondary metabolism.phenylpropanoids
solyc11g013740	7.1	S	S	S	1.2			signalling.G-proteins
solyc11g013830	7.3	S	S					signalling. phosphinositides.phosphatidylinositol-4-phosphate 5-kinase
solyc11g013880	7.3	S	S					signalling.receptor kinases.S-locus glycoprotein like
solyc11g016930	7.6	SI	SI					signalling.receptor kinases.leucine rich repeat X
solyc11g017040	7.8	S	S					protein.degradation.cysteine protease
solyc11g017070	7.9	S	S					hormone metabolism.brassinosteroid.signal transduction.other
solyc11g017270	8.1	SD	SD					signalling.receptor kinases.leucine rich repeat X
solyc11g017300	8.2	S	S					signalling.light.COP9 signalosome
solyc11g017335	8.2	SD	SD					protein.degradation.ubiquitin.E3.SCF.FBOX
solyc11g018550	8.7	S	S					redox.ascorbate and glutathione.ascorbate
solyc11g018670	8.9	S	S					stress.abiotic.heat
solyc11g018775	9.5				−2.5		−3.2	misc.glutathione S transferases
solyc11g018777	9.5				−2.3		−3.2	misc.glutathione S transferases
solyc11g018800	9.6	S	S		−2.9		−2.7	misc.glutathione S transferases
solyc11g018805	9.6				−2.3		−3.2	misc.glutathione S transferases
solyc11g019920	9.8	S	S					redox.thioredoxin.PDIL
solyc11g020040	10.0	I	I					stress.abiotic.heat
solyc11g020230	10.6	S	S				−1.2	signalling.receptor kinases.crinkly like
solyc11g020280	10.7	S	S					signalling.receptor kinases.leucine rich repeat XI
solyc11g022380	13.7	S	S	S				signalling.phosphinositides
solyc11g027810	19.1	S	S					protein.degradation.ubiquitin.E3.RING
solyc11g030730	23.2	S	S					secondary metabolism.flavonoids.dihydroflavonols.flavonoid 3’’-monooxygenase
solyc11g032220	26.1	SI	SI	SI	−3.7			hormone metabolism.jasmonate.synthesis-degradation.12-Oxo-PDA-reductase
solyc11g032225	26.1	S	?		−4.7			hormone metabolism.jasmonate.synthesis-degradation.12-Oxo-PDA-reductase
solyc11g033270	26.5	S	S					signalling.MAP kinases
solyc11g040040	40.3	S	S					signalling.light
solyc11g040050	40.2	S	S					signalling.G-proteins
solyc11g040340	38.1	S	S					cell wall.degradation.cellulases and beta -1,4-glucanases
solyc11g042930	34.2	S	S					protein.degradation.ubiquitin.E3.SCF.SKP
solyc11g043130	33.8	S	S					signalling.phosphinositides
solyc11g044310	33.3	S	S	S				protein.degradation
solyc11g044450	33.0				−1.2			stress.abiotic.heat
solyc11g044560	32.7	S	S					hormone metabolism.abscisic acid.signal transduction
solyc11g044910	32.3	SD	SD		−2.0			cell wall.degradation.mannan-xylose-arabinose-fucose
solyc11g044940	32.2	S	S					signalling.receptor kinases.crinkly like
solyc11g045240	31.6	S	S					protein.degradation
solyc11g045520	30.6	S	S		−1.2			hormone metabolism.ethylene.synthesis-degradation
solyc11g056680	47.7	S	S					signalling.receptor kinases.leucine rich repeat XI
solyc11g062260	49.4	S	S					protein.degradation.ubiquitin.E3.RING
solyc11g062430	49.8	S	S					signalling.light.COP9 signalosome
solyc11g062440	49.8	S	S					redox.ascorbate and glutathione.ascorbate
solyc11g064790	50.2	S	S					protein.degradation
solyc11g064830	50.3	SD	SD	D				protein.degradation.ubiquitin.E3.RING
solyc11g064835	50.4	I	I					hormone metabolism.ethylene.signal transduction
solyc11g064880	50.4	S	S					signalling.G-proteins
solyc11g064950	50.6	S	S					RNA.regulation of transcription.bZIP transcription factor family
solyc11g064953	50.6	S	S					RNA.regulation of transcription.bZIP transcription factor family
solyc11g065000	50.6	SD	SD					protein.degradation.ubiquitin.E3.SCF.FBOX
solyc11g065190	50.8	S	S					protein.degradation.ubiquitin.E2
solyc11g065210	50.9	S	S					protein.degradation.ubiquitin.ubiquitin protease
solyc11g065600	51.4	S	S					cell wall.modification
solyc11g065660	51.5	S	S					signalling.calcium
solyc11g065970	51.9				1.3			secondary metabolism.simple phenols
solyc11g066040	52.0	I	I	I				protein.degradation.ubiquitin.E3.RING
solyc11g066050	52.0	S	S					RNA.regulation of transcription.C2C2(Zn) DOF zinc finger family
solyc11g066060	52.0	S	S				1.4	stress.abiotic.heat
solyc11g066090	52.1	S	S					stress.abiotic
solyc11g066100	52.1				1.2			stress.abiotic.heat
solyc11g066150	52.1	S	S	S				cell wall.precursor synthesis.UXS
solyc11g066250	52.3	S	S				−1.1	protein.degradation
solyc11g066270	52.3	SI	SI	SD			−1.3	cell wall.modification
solyc11g066320	52.3	S	S					cell wall.hemicellulose synthesis.glucuronoxylan
solyc11g066510	52.6	S	S					protein.degradation.ubiquitin.E3.RING
solyc11g066580	52.6				3.0	1.7		secondary metabolism.flavonoids.dihydroflavonols.flavonoid 3″-monooxygenase
solyc11g066590	52.7	S	S					protein.degradation
solyc11g066670	52.7	S	S	S	1.2			secondary metabolism.phenylpropanoids.lignin biosynthesis
solyc11g066720	52.8	S	S					cell wall.precursor synthesis.AXS
solyc11g066730	52.8	SD	SD					signalling.light
solyc11g066780	52.9	S	S	S				protein.degradation
solyc11g066820	52.9	SI	SI	I				cell wall.cellulose synthesis
solyc11g068440	53.5	DI	DI	I				misc.beta 1,3 glucan hydrolases.glucan endo-1,3-beta-glucosidase
solyc11g068660	53.6	S	S					protein.degradation.cysteine protease
solyc11g068710	53.6	I	I	I	−1.2		−1.7	protein.degradation.ubiquitin.E3.SCF.FBOX
solyc11g069010	53.9	S	S					cell wall.degradation.mannan-xylose-arabinose-fucose
solyc11g069020	53.9				−2.0			stress.biotic.PR-proteins (Rx4)
solyc11g069050	54.0	S	S					secondary metabolism.phenylpropanoids.lignin biosynthesis.4CL
solyc11g069400	54.3	S	S					redox.thioredoxin.PDIL
solyc11g069600	54.5	S	S					protein.degradation.ubiquitin.E3.RING
solyc11g069620	54.6	S	S					stress.biotic.PR-proteins
solyc11g069660	54.5	S	S					stress.biotic.PR-proteins
solyc11g069800	54.7	S	S					hormone metabolism.jasmonate.synthesis-degradation.allene oxidase synthase
solyc11g069810	54.7	S	S					protein.degradation.cysteine protease
solyc11g069925	54.8	SI	SI	I				stress.biotic.PR-proteins
solyc11g069950	54.9	S	S					protein.degradation.metalloprotease
solyc11g069960	54.9	S	S				1.8	signalling.receptor kinases.leucine rich repeat III
solyc11g069990	55.0	S	S					stress.biotic.PR-proteins
solyc11g071340	55.1	S	S					cell wall.cell wall proteins.HRGP
solyc11g071423	55.2	S	S					stress.biotic.PR-proteins
solyc11g071500	55.3	S	S					RNA.regulation of transcription.MYB-related transcription factor
solyc11g071520	55.3	S	S					misc.beta 1,3 glucan hydrolases.glucan endo-1,3-beta-glucosidase
solyc11g071600	55.3	S	S		−1.3			hormone metabolism.abscisic acid.synthesis-degradation
solyc11g071610	55.3	S	S	S				hormone metabolism.abscisic acid.synthesis-degradation
solyc11g071620	55.3	S	S					hormone metabolism.abscisic acid.synthesis-degradation
solyc11g071640	55.4	S	SD					cell wall.degradation.cellulases and beta -1,4-glucanases
solyc11g071700	55.4	SD	SD					protein.degradation.ubiquitin
solyc11g071840	55.5	S	S	S				signalling.calcium
solyc11g071870	55.5	S	S	S				protein.degradation.ubiquitin.E2
solyc11g071910	55.5	D	D					signalling.G-proteins
solyc11g071920	55.5	S	S	S				protein.degradation.ubiquitin.E3.SCF.FBOX
solyc11g071930	55.5	S	S					stress.abiotic.heat
solyc11g072050	55.6	S	S					protein.degradation.ubiquitin.E3.SCF.FBOX
solyc11g072070	55.6	S	S					protein.degradation.ubiquitin.E3.BTB/POZ Cullin3.BTB/POZ
solyc11g072290	55.8	S	S	S				stress.biotic.signalling
solyc11g072540	56.0	S	S					protein.degradation.ubiquitin.E3.unspecified
solyc11g072590	56.1	S	S					protein.degradation.ubiquitin.E3.RING
solyc11g073120	56.5	I	I	I				RNA.regulation of transcription.MYB domain transcription factor

## Supplementary Materials

Supplementary materials can be found at https://www.mdpi.com/1422-0067/21/11/4070/s1. Table S1. DEGs identified between inoculated and mock sample for each tomato lines. Table S2. Enriched GO terms. Table S3. SNP/INDELs across all chromosomes.
