# Supplementary material for: Transcriptome-Based Analysis of Tomato Genotypes Resistant to Bacterial Spot (Xanthomonas perforans) Race T4
Source: Int J Mol Sci. 2020 Jun 6;21(11):4070. doi: 10.3390/ijms21114070 (PMC7313073; doi:10.3390/ijms21114070)
Supplement: Supplementary file 1 [file ijms-21-04070-s001.zip › Table S2 GOs.docx]

**Table X. GO enrichment DEGs**

Note: P, F and C are ontology abbreviation of biological process, molecular function and cellular component.

| GO term | Ontology | Description | N0. in input list | N0. in BG/Ref | p-value | FDR |
| --- | --- | --- | --- | --- | --- | --- |
| Up-regulated DEG for PI270443 | | | | | | |
| GO:0006323 | P | DNA packaging | [16](http://systemsbiology.cau.edu.cn/agriGOv2/termDetail.php?session=132042301.1&GO=GO:0006323) | 52 | 2.10E-17 | 1.10E-14 |
| GO:0006334 | P | nucleosome assembly | [15](http://systemsbiology.cau.edu.cn/agriGOv2/termDetail.php?session=132042301.1&GO=GO:0006334) | 47 | 1.40E-16 | 2.40E-14 |
| GO:0034728 | P | nucleosome organization | [15](http://systemsbiology.cau.edu.cn/agriGOv2/termDetail.php?session=132042301.1&GO=GO:0034728) | 48 | 1.80E-16 | 2.40E-14 |
| GO:0031497 | P | chromatin assembly | [15](http://systemsbiology.cau.edu.cn/agriGOv2/termDetail.php?session=132042301.1&GO=GO:0031497) | 47 | 1.40E-16 | 2.40E-14 |
| GO:0065004 | P | protein-DNA complex assembly | [15](http://systemsbiology.cau.edu.cn/agriGOv2/termDetail.php?session=132042301.1&GO=GO:0065004) | 50 | 2.90E-16 | 2.70E-14 |
| GO:0006333 | P | chromatin assembly or disassembly | [15](http://systemsbiology.cau.edu.cn/agriGOv2/termDetail.php?session=132042301.1&GO=GO:0006333) | 50 | 2.90E-16 | 2.70E-14 |
| GO:0071824 | P | protein-DNA complex subunit organization | [15](http://systemsbiology.cau.edu.cn/agriGOv2/termDetail.php?session=132042301.1&GO=GO:0071824) | 51 | 3.80E-16 | 2.90E-14 |
| GO:0071103 | P | DNA conformation change | [16](http://systemsbiology.cau.edu.cn/agriGOv2/termDetail.php?session=132042301.1&GO=GO:0071103) | 66 | 5.20E-16 | 3.50E-14 |
| GO:0009765 | P | photosynthesis, light harvesting | [13](http://systemsbiology.cau.edu.cn/agriGOv2/termDetail.php?session=132042301.1&GO=GO:0009765) | 35 | 3.00E-15 | 1.80E-13 |
| GO:0006325 | P | chromatin organization | [15](http://systemsbiology.cau.edu.cn/agriGOv2/termDetail.php?session=132042301.1&GO=GO:0006325) | 114 | 1.10E-11 | 6.20E-10 |
| GO:0019684 | P | photosynthesis, light reaction | [13](http://systemsbiology.cau.edu.cn/agriGOv2/termDetail.php?session=132042301.1&GO=GO:0019684) | 86 | 5.90E-11 | 2.90E-09 |
| GO:0070271 | P | protein complex biogenesis | [15](http://systemsbiology.cau.edu.cn/agriGOv2/termDetail.php?session=132042301.1&GO=GO:0070271) | 141 | 1.80E-10 | 7.40E-09 |
| GO:0006461 | P | protein complex assembly | [15](http://systemsbiology.cau.edu.cn/agriGOv2/termDetail.php?session=132042301.1&GO=GO:0006461) | 141 | 1.80E-10 | 7.40E-09 |
| GO:0051276 | P | chromosome organization | [18](http://systemsbiology.cau.edu.cn/agriGOv2/termDetail.php?session=132042301.1&GO=GO:0051276) | 228 | 2.40E-10 | 9.30E-09 |
| GO:0034622 | P | cellular macromolecular complex assembly | [15](http://systemsbiology.cau.edu.cn/agriGOv2/termDetail.php?session=132042301.1&GO=GO:0034622) | 153 | 5.10E-10 | 1.80E-08 |
| GO:0065003 | P | macromolecular complex assembly | [15](http://systemsbiology.cau.edu.cn/agriGOv2/termDetail.php?session=132042301.1&GO=GO:0065003) | 167 | 1.50E-09 | 5.30E-08 |
| GO:0071822 | P | protein complex subunit organization | [15](http://systemsbiology.cau.edu.cn/agriGOv2/termDetail.php?session=132042301.1&GO=GO:0071822) | 170 | 1.90E-09 | 6.20E-08 |
| GO:0022607 | P | cellular component assembly | [16](http://systemsbiology.cau.edu.cn/agriGOv2/termDetail.php?session=132042301.1&GO=GO:0022607) | 230 | 1.30E-08 | 3.90E-07 |
| GO:0006996 | P | organelle organization | [20](http://systemsbiology.cau.edu.cn/agriGOv2/termDetail.php?session=132042301.1&GO=GO:0006996) | 374 | 1.40E-08 | 3.90E-07 |
| GO:0015979 | P | photosynthesis | [13](http://systemsbiology.cau.edu.cn/agriGOv2/termDetail.php?session=132042301.1&GO=GO:0015979) | 178 | 1.90E-07 | 5.10E-06 |
| GO:0044085 | P | cellular component biogenesis | [17](http://systemsbiology.cau.edu.cn/agriGOv2/termDetail.php?session=132042301.1&GO=GO:0044085) | 337 | 3.80E-07 | 9.90E-06 |
| GO:0043933 | P | macromolecular complex subunit organization | [15](http://systemsbiology.cau.edu.cn/agriGOv2/termDetail.php?session=132042301.1&GO=GO:0043933) | 263 | 4.40E-07 | 1.10E-05 |
| GO:0006091 | P | generation of precursor metabolites and energy | [13](http://systemsbiology.cau.edu.cn/agriGOv2/termDetail.php?session=132042301.1&GO=GO:0006091) | 246 | 5.70E-06 | 0.00014 |
| GO:0016043 | P | cellular component organization | [20](http://systemsbiology.cau.edu.cn/agriGOv2/termDetail.php?session=132042301.1&GO=GO:0016043) | 691 | 0.00011 | 0.0025 |
| GO:0071840 | P | cellular component organization or biogenesis | [21](http://systemsbiology.cau.edu.cn/agriGOv2/termDetail.php?session=132042301.1&GO=GO:0071840) | 798 | 0.00026 | 0.0057 |
| GO:0046982 | F | protein heterodimerization activity | [32](http://systemsbiology.cau.edu.cn/agriGOv2/termDetail.php?session=132042301.1&GO=GO:0046982) | 150 | 1.80E-29 | 4.00E-27 |
| GO:0046983 | F | protein dimerization activity | [36](http://systemsbiology.cau.edu.cn/agriGOv2/termDetail.php?session=132042301.1&GO=GO:0046983) | 680 | 1.60E-14 | 1.80E-12 |
| GO:0003677 | F | DNA binding | [50](http://systemsbiology.cau.edu.cn/agriGOv2/termDetail.php?session=132042301.1&GO=GO:0003677) | 1915 | 9.50E-09 | 7.10E-07 |
| GO:0005515 | F | protein binding | [73](http://systemsbiology.cau.edu.cn/agriGOv2/termDetail.php?session=132042301.1&GO=GO:0005515) | 4493 | 0.00021 | 0.012 |
| GO:0003676 | F | nucleic acid binding | [53](http://systemsbiology.cau.edu.cn/agriGOv2/termDetail.php?session=132042301.1&GO=GO:0003676) | 3148 | 0.00097 | 0.044 |
| GO:0044815 | C | DNA packaging complex | [37](http://systemsbiology.cau.edu.cn/agriGOv2/termDetail.php?session=132042301.1&GO=GO:0044815) | 103 | 4.20E-41 | 2.20E-39 |
| GO:0000786 | C | nucleosome | [37](http://systemsbiology.cau.edu.cn/agriGOv2/termDetail.php?session=132042301.1&GO=GO:0000786) | 101 | 2.30E-41 | 2.20E-39 |
| GO:0032993 | C | protein-DNA complex | [37](http://systemsbiology.cau.edu.cn/agriGOv2/termDetail.php?session=132042301.1&GO=GO:0032993) | 105 | 7.50E-41 | 2.60E-39 |
| GO:0000785 | C | chromatin | [37](http://systemsbiology.cau.edu.cn/agriGOv2/termDetail.php?session=132042301.1&GO=GO:0000785) | 118 | 2.80E-39 | 7.20E-38 |
| GO:0005694 | C | chromosome | [39](http://systemsbiology.cau.edu.cn/agriGOv2/termDetail.php?session=132042301.1&GO=GO:0005694) | 174 | 1.40E-36 | 3.00E-35 |
| GO:0044427 | C | chromosomal part | [37](http://systemsbiology.cau.edu.cn/agriGOv2/termDetail.php?session=132042301.1&GO=GO:0044427) | 156 | 1.80E-35 | 3.10E-34 |
| GO:0043232 | C | intracellular non-membrane-bounded organelle | [46](http://systemsbiology.cau.edu.cn/agriGOv2/termDetail.php?session=132042301.1&GO=GO:0043232) | 764 | 1.50E-20 | 2.00E-19 |
| GO:0043228 | C | non-membrane-bounded organelle | [46](http://systemsbiology.cau.edu.cn/agriGOv2/termDetail.php?session=132042301.1&GO=GO:0043228) | 764 | 1.50E-20 | 2.00E-19 |
| GO:0044422 | C | organelle part | [45](http://systemsbiology.cau.edu.cn/agriGOv2/termDetail.php?session=132042301.1&GO=GO:0044422) | 735 | 2.30E-20 | 2.40E-19 |
| GO:0044446 | C | intracellular organelle part | [45](http://systemsbiology.cau.edu.cn/agriGOv2/termDetail.php?session=132042301.1&GO=GO:0044446) | 735 | 2.30E-20 | 2.40E-19 |
| GO:0043229 | C | intracellular organelle | [68](http://systemsbiology.cau.edu.cn/agriGOv2/termDetail.php?session=132042301.1&GO=GO:0043229) | 1905 | 1.90E-18 | 1.60E-17 |
| GO:0043226 | C | organelle | [68](http://systemsbiology.cau.edu.cn/agriGOv2/termDetail.php?session=132042301.1&GO=GO:0043226) | 1905 | 1.90E-18 | 1.60E-17 |
| GO:0005634 | C | nucleus | [42](http://systemsbiology.cau.edu.cn/agriGOv2/termDetail.php?session=132042301.1&GO=GO:0005634) | 859 | 1.10E-15 | 8.60E-15 |
| GO:0043234 | C | protein complex | [42](http://systemsbiology.cau.edu.cn/agriGOv2/termDetail.php?session=132042301.1&GO=GO:0043234) | 901 | 5.10E-15 | 3.90E-14 |
| GO:0044424 | C | intracellular part | [71](http://systemsbiology.cau.edu.cn/agriGOv2/termDetail.php?session=132042301.1&GO=GO:0044424) | 2587 | 1.70E-13 | 1.20E-12 |
| GO:0005622 | C | intracellular | [72](http://systemsbiology.cau.edu.cn/agriGOv2/termDetail.php?session=132042301.1&GO=GO:0005622) | 2728 | 7.00E-13 | 4.60E-12 |
| GO:0044464 | C | cell part | [73](http://systemsbiology.cau.edu.cn/agriGOv2/termDetail.php?session=132042301.1&GO=GO:0044464) | 2892 | 3.90E-12 | 2.30E-11 |
| GO:0005623 | C | cell | [73](http://systemsbiology.cau.edu.cn/agriGOv2/termDetail.php?session=132042301.1&GO=GO:0005623) | 2892 | 3.90E-12 | 2.30E-11 |
| GO:0043231 | C | intracellular membrane-bounded organelle | [45](http://systemsbiology.cau.edu.cn/agriGOv2/termDetail.php?session=132042301.1&GO=GO:0043231) | 1271 | 5.40E-12 | 2.80E-11 |
| GO:0043227 | C | membrane-bounded organelle | [45](http://systemsbiology.cau.edu.cn/agriGOv2/termDetail.php?session=132042301.1&GO=GO:0043227) | 1271 | 5.40E-12 | 2.80E-11 |
| GO:0032991 | C | macromolecular complex | [46](http://systemsbiology.cau.edu.cn/agriGOv2/termDetail.php?session=132042301.1&GO=GO:0032991) | 1429 | 6.80E-11 | 3.40E-10 |
| Down-regulated DEG for PI270443 after inoculation | | | | | | |
| GO:0016410 | F | N-acyltransferase activity | [7](http://systemsbiology.cau.edu.cn/agriGOv2/termDetail.php?session=569451897.1&GO=GO:0016410) | 67 | 0.00011 | 0.01 |
| GO:0001071 | F | nucleic acid binding transcription factor activity | [22](http://systemsbiology.cau.edu.cn/agriGOv2/termDetail.php?session=569451897.1&GO=GO:0001071) | 570 | 6.70E-05 | 0.01 |
| GO:0008080 | F | N-acetyltransferase activity | [7](http://systemsbiology.cau.edu.cn/agriGOv2/termDetail.php?session=569451897.1&GO=GO:0008080) | 64 | 8.10E-05 | 0.01 |
| GO:0003700 | F | transcription factor activity, sequence-specific DNA binding | [22](http://systemsbiology.cau.edu.cn/agriGOv2/termDetail.php?session=569451897.1&GO=GO:0003700) | 570 | 6.70E-05 | 0.01 |
| GO:0016407 | F | acetyltransferase activity | [7](http://systemsbiology.cau.edu.cn/agriGOv2/termDetail.php?session=569451897.1&GO=GO:0016407) | 70 | 0.00014 | 0.011 |
| GO:0016798 | F | hydrolase activity, acting on glycosyl bonds | [17](http://systemsbiology.cau.edu.cn/agriGOv2/termDetail.php?session=569451897.1&GO=GO:0016798) | 416 | 0.00024 | 0.016 |
| GO:0004252 | F | serine-type endopeptidase activity | [9](http://systemsbiology.cau.edu.cn/agriGOv2/termDetail.php?session=569451897.1&GO=GO:0004252) | 155 | 0.00072 | 0.04 |
| GO:0017171 | F | serine hydrolase activity | [11](http://systemsbiology.cau.edu.cn/agriGOv2/termDetail.php?session=569451897.1&GO=GO:0017171) | 239 | 0.0012 | 0.044 |
| GO:0004553 | F | hydrolase activity, hydrolyzing O-glycosyl compounds | [15](http://systemsbiology.cau.edu.cn/agriGOv2/termDetail.php?session=569451897.1&GO=GO:0004553) | 394 | 0.0011 | 0.044 |
| GO:0008236 | F | serine-type peptidase activity | [11](http://systemsbiology.cau.edu.cn/agriGOv2/termDetail.php?session=569451897.1&GO=GO:0008236) | 239 | 0.0012 | 0.044 |
| GO:0003824 | F | catalytic activity | [153](http://systemsbiology.cau.edu.cn/agriGOv2/termDetail.php?session=569451897.1&GO=GO:0003824) | 8492 | 0.0012 | 0.044 |

| GO term | Ontology | Description | N0. in input list | N0. in BG/Ref | p-value | FDR |
| --- | --- | --- | --- | --- | --- | --- |
| Up-regulated DEG for NC1CELBR after inoculation | | | | | | |
| GO:0016758 | F | transferase activity, transferring hexosyl groups | [9](http://systemsbiology.cau.edu.cn/agriGOv2/termDetail.php?session=818803392.1&GO=GO:0016758) | 467 | 1.90E-06 | 8.80E-05 |
| GO:0016757 | F | transferase activity, transferring glycosyl groups | [9](http://systemsbiology.cau.edu.cn/agriGOv2/termDetail.php?session=818803392.1&GO=GO:0016757) | 557 | 7.90E-06 | 0.00018 |
| Down-regulated DEG for NC1CELBR after inoculation | | | | | | |
| GO:0044248 | P | cellular catabolic process | [7](http://systemsbiology.cau.edu.cn/agriGOv2/termDetail.php?session=428803544.1&GO=GO:0044248) | 421 | 0.00031 | 0.049 |
| GO:0006952 | P | defense response | [5](http://systemsbiology.cau.edu.cn/agriGOv2/termDetail.php?session=428803544.1&GO=GO:0006952) | 166 | 0.00017 | 0.049 |
| GO:0004553 | F | hydrolase activity, hydrolyzing O-glycosyl compounds | [9](http://systemsbiology.cau.edu.cn/agriGOv2/termDetail.php?session=428803544.1&GO=GO:0004553) | 394 | 3.30E-06 | 0.00023 |
| GO:0016798 | F | hydrolase activity, acting on glycosyl bonds | [9](http://systemsbiology.cau.edu.cn/agriGOv2/termDetail.php?session=428803544.1&GO=GO:0016798) | 416 | 5.10E-06 | 0.00023 |
| GO:0001071 | F | nucleic acid binding transcription factor activity | [7](http://systemsbiology.cau.edu.cn/agriGOv2/termDetail.php?session=428803544.1&GO=GO:0001071) | 570 | 0.0018 | 0.04 |
| GO:0003700 | F | transcription factor activity, sequence-specific DNA binding | [7](http://systemsbiology.cau.edu.cn/agriGOv2/termDetail.php?session=428803544.1&GO=GO:0003700) | 570 | 0.0018 | 0.04 |
| GO:0016021 | C | integral component of membrane | [9](http://systemsbiology.cau.edu.cn/agriGOv2/termDetail.php?session=428803544.1&GO=GO:0016021) | 1085 | 0.0055 | 0.022 |
| GO:0031224 | C | intrinsic component of membrane | [9](http://systemsbiology.cau.edu.cn/agriGOv2/termDetail.php?session=428803544.1&GO=GO:0031224) | 1108 | 0.0063 | 0.022 |
| GO:0044425 | C | membrane part | [10](http://systemsbiology.cau.edu.cn/agriGOv2/termDetail.php?session=428803544.1&GO=GO:0044425) | 1360 | 0.0078 | 0.022 |

| GO term | Ontology | Description | No. in input list | N0. in BG/Ref | p-value | FDR |
| --- | --- | --- | --- | --- | --- | --- |
| Up-regulated DEG for NC714 after inoculation | | | | | | |
| GO:0046982 | F | protein heterodimerization activity | [10](http://systemsbiology.cau.edu.cn/agriGOv2/termDetail.php?session=610668310.1&GO=GO:0046982) | 150 | 1.10E-11 | 1.20E-09 |
| GO:0046983 | F | protein dimerization activity | [12](http://systemsbiology.cau.edu.cn/agriGOv2/termDetail.php?session=610668310.1&GO=GO:0046983) | 680 | 1.90E-07 | 9.70E-06 |
| GO:0003677 | F | DNA binding | [14](http://systemsbiology.cau.edu.cn/agriGOv2/termDetail.php?session=610668310.1&GO=GO:0003677) | 1915 | 0.00036 | 0.012 |
| GO:0044815 | C | DNA packaging complex | [10](http://systemsbiology.cau.edu.cn/agriGOv2/termDetail.php?session=610668310.1&GO=GO:0044815) | 103 | 3.40E-13 | 1.00E-11 |
| GO:0000786 | C | nucleosome | [10](http://systemsbiology.cau.edu.cn/agriGOv2/termDetail.php?session=610668310.1&GO=GO:0000786) | 101 | 2.80E-13 | 1.00E-11 |
| GO:0032993 | C | protein-DNA complex | [10](http://systemsbiology.cau.edu.cn/agriGOv2/termDetail.php?session=610668310.1&GO=GO:0032993) | 105 | 4.00E-13 | 1.00E-11 |
| GO:0000785 | C | chromatin | [10](http://systemsbiology.cau.edu.cn/agriGOv2/termDetail.php?session=610668310.1&GO=GO:0000785) | 118 | 1.20E-12 | 2.30E-11 |
| GO:0044427 | C | chromosomal part | [10](http://systemsbiology.cau.edu.cn/agriGOv2/termDetail.php?session=610668310.1&GO=GO:0044427) | 156 | 1.60E-11 | 2.50E-10 |
| GO:0005694 | C | chromosome | [10](http://systemsbiology.cau.edu.cn/agriGOv2/termDetail.php?session=610668310.1&GO=GO:0005694) | 174 | 4.50E-11 | 5.70E-10 |
| GO:0044422 | C | organelle part | [10](http://systemsbiology.cau.edu.cn/agriGOv2/termDetail.php?session=610668310.1&GO=GO:0044422) | 735 | 2.10E-05 | 0.0002 |
| GO:0044446 | C | intracellular organelle part | [10](http://systemsbiology.cau.edu.cn/agriGOv2/termDetail.php?session=610668310.1&GO=GO:0044446) | 735 | 2.10E-05 | 0.0002 |
| GO:0043232 | C | intracellular non-membrane-bounded organelle | [10](http://systemsbiology.cau.edu.cn/agriGOv2/termDetail.php?session=610668310.1&GO=GO:0043232) | 764 | 3.00E-05 | 0.00023 |
| GO:0043228 | C | non-membrane-bounded organelle | [10](http://systemsbiology.cau.edu.cn/agriGOv2/termDetail.php?session=610668310.1&GO=GO:0043228) | 764 | 3.00E-05 | 0.00023 |
| GO:0044464 | C | cell part | [19](http://systemsbiology.cau.edu.cn/agriGOv2/termDetail.php?session=610668310.1&GO=GO:0044464) | 2892 | 9.10E-05 | 0.00058 |
| GO:0005623 | C | cell | [19](http://systemsbiology.cau.edu.cn/agriGOv2/termDetail.php?session=610668310.1&GO=GO:0005623) | 2892 | 9.10E-05 | 0.00058 |
| GO:0043234 | C | protein complex | [10](http://systemsbiology.cau.edu.cn/agriGOv2/termDetail.php?session=610668310.1&GO=GO:0043234) | 901 | 0.00012 | 0.00068 |
| GO:0005622 | C | intracellular | [18](http://systemsbiology.cau.edu.cn/agriGOv2/termDetail.php?session=610668310.1&GO=GO:0005622) | 2728 | 0.00014 | 0.00078 |
| GO:0044424 | C | intracellular part | [17](http://systemsbiology.cau.edu.cn/agriGOv2/termDetail.php?session=610668310.1&GO=GO:0044424) | 2587 | 0.00025 | 0.0013 |
| GO:0043229 | C | intracellular organelle | [14](http://systemsbiology.cau.edu.cn/agriGOv2/termDetail.php?session=610668310.1&GO=GO:0043229) | 1905 | 0.00034 | 0.0015 |
| GO:0043226 | C | organelle | [14](http://systemsbiology.cau.edu.cn/agriGOv2/termDetail.php?session=610668310.1&GO=GO:0043226) | 1905 | 0.00034 | 0.0015 |
| GO:0032991 | C | macromolecular complex | [10](http://systemsbiology.cau.edu.cn/agriGOv2/termDetail.php?session=610668310.1&GO=GO:0032991) | 1429 | 0.0039 | 0.017 |
| Down-regulated DEG for NC714 after inoculation | | | | | | |
| GO:2001141 | P | regulation of RNA biosynthetic process | [26](http://systemsbiology.cau.edu.cn/agriGOv2/termDetail.php?session=944978279.1&GO=GO:2001141) | 1082 | 2.40E-05 | 0.0015 |
| GO:0051704 | P | multi-organism process | [8](http://systemsbiology.cau.edu.cn/agriGOv2/termDetail.php?session=944978279.1&GO=GO:0051704) | 106 | 1.50E-05 | 0.0015 |
| GO:0009889 | P | regulation of biosynthetic process | [26](http://systemsbiology.cau.edu.cn/agriGOv2/termDetail.php?session=944978279.1&GO=GO:0009889) | 1096 | 3.00E-05 | 0.0015 |
| GO:0006355 | P | regulation of transcription, DNA-templated | [26](http://systemsbiology.cau.edu.cn/agriGOv2/termDetail.php?session=944978279.1&GO=GO:0006355) | 1082 | 2.40E-05 | 0.0015 |
| GO:0010556 | P | regulation of macromolecule biosynthetic process | [26](http://systemsbiology.cau.edu.cn/agriGOv2/termDetail.php?session=944978279.1&GO=GO:0010556) | 1095 | 3.00E-05 | 0.0015 |
| GO:1903506 | P | regulation of nucleic acid-templated transcription | [26](http://systemsbiology.cau.edu.cn/agriGOv2/termDetail.php?session=944978279.1&GO=GO:1903506) | 1082 | 2.40E-05 | 0.0015 |
| GO:0051252 | P | regulation of RNA metabolic process | [26](http://systemsbiology.cau.edu.cn/agriGOv2/termDetail.php?session=944978279.1&GO=GO:0051252) | 1084 | 2.50E-05 | 0.0015 |
| GO:0031326 | P | regulation of cellular biosynthetic process | [26](http://systemsbiology.cau.edu.cn/agriGOv2/termDetail.php?session=944978279.1&GO=GO:0031326) | 1096 | 3.00E-05 | 0.0015 |
| GO:2000112 | P | regulation of cellular macromolecule biosynthetic process | [26](http://systemsbiology.cau.edu.cn/agriGOv2/termDetail.php?session=944978279.1&GO=GO:2000112) | 1095 | 3.00E-05 | 0.0015 |
| GO:0019219 | P | regulation of nucleobase-containing compound metabolic process | [26](http://systemsbiology.cau.edu.cn/agriGOv2/termDetail.php?session=944978279.1&GO=GO:0019219) | 1095 | 3.00E-05 | 0.0015 |
| GO:0051171 | P | regulation of nitrogen compound metabolic process | [26](http://systemsbiology.cau.edu.cn/agriGOv2/termDetail.php?session=944978279.1&GO=GO:0051171) | 1106 | 3.50E-05 | 0.0016 |
| GO:0010468 | P | regulation of gene expression | [26](http://systemsbiology.cau.edu.cn/agriGOv2/termDetail.php?session=944978279.1&GO=GO:0010468) | 1117 | 4.10E-05 | 0.0018 |
| GO:0080090 | P | regulation of primary metabolic process | [26](http://systemsbiology.cau.edu.cn/agriGOv2/termDetail.php?session=944978279.1&GO=GO:0080090) | 1145 | 6.20E-05 | 0.0024 |
| GO:0031323 | P | regulation of cellular metabolic process | [26](http://systemsbiology.cau.edu.cn/agriGOv2/termDetail.php?session=944978279.1&GO=GO:0031323) | 1149 | 6.50E-05 | 0.0024 |
| GO:0019222 | P | regulation of metabolic process | [26](http://systemsbiology.cau.edu.cn/agriGOv2/termDetail.php?session=944978279.1&GO=GO:0019222) | 1181 | 0.0001 | 0.0026 |
| GO:0060255 | P | regulation of macromolecule metabolic process | [26](http://systemsbiology.cau.edu.cn/agriGOv2/termDetail.php?session=944978279.1&GO=GO:0060255) | 1166 | 8.30E-05 | 0.0026 |
| GO:0044036 | P | cell wall macromolecule metabolic process | [6](http://systemsbiology.cau.edu.cn/agriGOv2/termDetail.php?session=944978279.1&GO=GO:0044036) | 68 | 8.00E-05 | 0.0026 |
| GO:0042743 | P | hydrogen peroxide metabolic process | [7](http://systemsbiology.cau.edu.cn/agriGOv2/termDetail.php?session=944978279.1&GO=GO:0042743) | 104 | 0.0001 | 0.0026 |
| GO:0044248 | P | cellular catabolic process | [14](http://systemsbiology.cau.edu.cn/agriGOv2/termDetail.php?session=944978279.1&GO=GO:0044248) | 421 | 8.80E-05 | 0.0026 |
| GO:0042744 | P | hydrogen peroxide catabolic process | [7](http://systemsbiology.cau.edu.cn/agriGOv2/termDetail.php?session=944978279.1&GO=GO:0042744) | 104 | 0.0001 | 0.0026 |
| GO:0043170 | P | macromolecule metabolic process | [73](http://systemsbiology.cau.edu.cn/agriGOv2/termDetail.php?session=944978279.1&GO=GO:0043170) | 5077 | 0.00014 | 0.0034 |
| GO:0006950 | P | response to stress | [18](http://systemsbiology.cau.edu.cn/agriGOv2/termDetail.php?session=944978279.1&GO=GO:0006950) | 690 | 0.00018 | 0.0042 |
| GO:0097659 | P | nucleic acid-templated transcription | [26](http://systemsbiology.cau.edu.cn/agriGOv2/termDetail.php?session=944978279.1&GO=GO:0097659) | 1237 | 0.00021 | 0.0044 |
| GO:0006351 | P | transcription, DNA-templated | [26](http://systemsbiology.cau.edu.cn/agriGOv2/termDetail.php?session=944978279.1&GO=GO:0006351) | 1237 | 0.00021 | 0.0044 |
| GO:0032774 | P | RNA biosynthetic process | [26](http://systemsbiology.cau.edu.cn/agriGOv2/termDetail.php?session=944978279.1&GO=GO:0032774) | 1239 | 0.00021 | 0.0044 |
| GO:0072593 | P | reactive oxygen species metabolic process | [7](http://systemsbiology.cau.edu.cn/agriGOv2/termDetail.php?session=944978279.1&GO=GO:0072593) | 119 | 0.00022 | 0.0044 |
| GO:0009607 | P | response to biotic stimulus | [6](http://systemsbiology.cau.edu.cn/agriGOv2/termDetail.php?session=944978279.1&GO=GO:0009607) | 87 | 0.00028 | 0.0054 |
| GO:0006468 | P | protein phosphorylation | [26](http://systemsbiology.cau.edu.cn/agriGOv2/termDetail.php?session=944978279.1&GO=GO:0006468) | 1270 | 0.00031 | 0.0057 |
| GO:0071704 | P | organic substance metabolic process | [89](http://systemsbiology.cau.edu.cn/agriGOv2/termDetail.php?session=944978279.1&GO=GO:0071704) | 6832 | 0.00059 | 0.01 |
| GO:0044706 | P | multi-multicellular organism process | [5](http://systemsbiology.cau.edu.cn/agriGOv2/termDetail.php?session=944978279.1&GO=GO:0044706) | 68 | 0.0007 | 0.011 |
| GO:0009875 | P | pollen-pistil interaction | [5](http://systemsbiology.cau.edu.cn/agriGOv2/termDetail.php?session=944978279.1&GO=GO:0009875) | 68 | 0.0007 | 0.011 |
| GO:0008037 | P | cell recognition | [5](http://systemsbiology.cau.edu.cn/agriGOv2/termDetail.php?session=944978279.1&GO=GO:0008037) | 68 | 0.0007 | 0.011 |
| GO:0048544 | P | recognition of pollen | [5](http://systemsbiology.cau.edu.cn/agriGOv2/termDetail.php?session=944978279.1&GO=GO:0048544) | 68 | 0.0007 | 0.011 |
| GO:0009856 | P | pollination | [5](http://systemsbiology.cau.edu.cn/agriGOv2/termDetail.php?session=944978279.1&GO=GO:0009856) | 68 | 0.0007 | 0.011 |
| GO:0050794 | P | regulation of cellular process | [31](http://systemsbiology.cau.edu.cn/agriGOv2/termDetail.php?session=944978279.1&GO=GO:0050794) | 1732 | 0.00077 | 0.011 |
| GO:0009056 | P | catabolic process | [14](http://systemsbiology.cau.edu.cn/agriGOv2/termDetail.php?session=944978279.1&GO=GO:0009056) | 530 | 0.00084 | 0.012 |
| GO:0034654 | P | nucleobase-containing compound biosynthetic process | [26](http://systemsbiology.cau.edu.cn/agriGOv2/termDetail.php?session=944978279.1&GO=GO:0034654) | 1377 | 0.001 | 0.014 |
| GO:0044238 | P | primary metabolic process | [84](http://systemsbiology.cau.edu.cn/agriGOv2/termDetail.php?session=944978279.1&GO=GO:0044238) | 6456 | 0.001 | 0.014 |
| GO:0044703 | P | multi-organism reproductive process | [5](http://systemsbiology.cau.edu.cn/agriGOv2/termDetail.php?session=944978279.1&GO=GO:0044703) | 76 | 0.0011 | 0.014 |
| GO:0006979 | P | response to oxidative stress | [7](http://systemsbiology.cau.edu.cn/agriGOv2/termDetail.php?session=944978279.1&GO=GO:0006979) | 158 | 0.0011 | 0.014 |
| GO:0018130 | P | heterocycle biosynthetic process | [27](http://systemsbiology.cau.edu.cn/agriGOv2/termDetail.php?session=944978279.1&GO=GO:0018130) | 1472 | 0.0012 | 0.015 |
| GO:0050789 | P | regulation of biological process | [31](http://systemsbiology.cau.edu.cn/agriGOv2/termDetail.php?session=944978279.1&GO=GO:0050789) | 1792 | 0.0013 | 0.016 |
| GO:0008152 | P | metabolic process | [110](http://systemsbiology.cau.edu.cn/agriGOv2/termDetail.php?session=944978279.1&GO=GO:0008152) | 9091 | 0.0013 | 0.016 |
| GO:0016310 | P | phosphorylation | [26](http://systemsbiology.cau.edu.cn/agriGOv2/termDetail.php?session=944978279.1&GO=GO:0016310) | 1403 | 0.0013 | 0.016 |
| GO:0044702 | P | single organism reproductive process | [5](http://systemsbiology.cau.edu.cn/agriGOv2/termDetail.php?session=944978279.1&GO=GO:0044702) | 82 | 0.0015 | 0.018 |
| GO:1901362 | P | organic cyclic compound biosynthetic process | [27](http://systemsbiology.cau.edu.cn/agriGOv2/termDetail.php?session=944978279.1&GO=GO:1901362) | 1514 | 0.0018 | 0.02 |
| GO:0044260 | P | cellular macromolecule metabolic process | [62](http://systemsbiology.cau.edu.cn/agriGOv2/termDetail.php?session=944978279.1&GO=GO:0044260) | 4501 | 0.0018 | 0.02 |
| GO:0044237 | P | cellular metabolic process | [78](http://systemsbiology.cau.edu.cn/agriGOv2/termDetail.php?session=944978279.1&GO=GO:0044237) | 6041 | 0.0023 | 0.024 |
| GO:0019438 | P | aromatic compound biosynthetic process | [26](http://systemsbiology.cau.edu.cn/agriGOv2/termDetail.php?session=944978279.1&GO=GO:0019438) | 1463 | 0.0023 | 0.024 |
| GO:0000003 | P | reproduction | [5](http://systemsbiology.cau.edu.cn/agriGOv2/termDetail.php?session=944978279.1&GO=GO:0000003) | 92 | 0.0025 | 0.025 |
| GO:0022414 | P | reproductive process | [5](http://systemsbiology.cau.edu.cn/agriGOv2/termDetail.php?session=944978279.1&GO=GO:0022414) | 92 | 0.0025 | 0.025 |
| GO:0065007 | P | biological regulation | [31](http://systemsbiology.cau.edu.cn/agriGOv2/termDetail.php?session=944978279.1&GO=GO:0065007) | 1927 | 0.004 | 0.039 |
| GO:0036211 | P | protein modification process | [29](http://systemsbiology.cau.edu.cn/agriGOv2/termDetail.php?session=944978279.1&GO=GO:0036211) | 1773 | 0.0043 | 0.041 |
| GO:0006464 | P | cellular protein modification process | [29](http://systemsbiology.cau.edu.cn/agriGOv2/termDetail.php?session=944978279.1&GO=GO:0006464) | 1773 | 0.0043 | 0.041 |
| GO:0001071 | F | nucleic acid binding transcription factor activity | [23](http://systemsbiology.cau.edu.cn/agriGOv2/termDetail.php?session=944978279.1&GO=GO:0001071) | 570 | 1.50E-08 | 2.10E-06 |
| GO:0003700 | F | transcription factor activity, sequence-specific DNA binding | [23](http://systemsbiology.cau.edu.cn/agriGOv2/termDetail.php?session=944978279.1&GO=GO:0003700) | 570 | 1.50E-08 | 2.10E-06 |
| GO:0008061 | F | chitin binding | [5](http://systemsbiology.cau.edu.cn/agriGOv2/termDetail.php?session=944978279.1&GO=GO:0008061) | 23 | 6.50E-06 | 0.00059 |
| GO:0004553 | F | hydrolase activity, hydrolyzing O-glycosyl compounds | [13](http://systemsbiology.cau.edu.cn/agriGOv2/termDetail.php?session=944978279.1&GO=GO:0004553) | 394 | 0.00017 | 0.011 |
| GO:0004672 | F | protein kinase activity | [26](http://systemsbiology.cau.edu.cn/agriGOv2/termDetail.php?session=944978279.1&GO=GO:0004672) | 1233 | 0.0002 | 0.011 |
| GO:0016798 | F | hydrolase activity, acting on glycosyl bonds | [13](http://systemsbiology.cau.edu.cn/agriGOv2/termDetail.php?session=944978279.1&GO=GO:0016798) | 416 | 0.00028 | 0.013 |
| GO:0004674 | F | protein serine/threonine kinase activity | [6](http://systemsbiology.cau.edu.cn/agriGOv2/termDetail.php?session=944978279.1&GO=GO:0004674) | 97 | 0.00049 | 0.017 |
| GO:0043565 | F | sequence-specific DNA binding | [12](http://systemsbiology.cau.edu.cn/agriGOv2/termDetail.php?session=944978279.1&GO=GO:0043565) | 385 | 0.00049 | 0.017 |
| GO:0016773 | F | phosphotransferase activity, alcohol group as acceptor | [26](http://systemsbiology.cau.edu.cn/agriGOv2/termDetail.php?session=944978279.1&GO=GO:0016773) | 1352 | 0.00078 | 0.024 |
| GO:0004601 | F | peroxidase activity | [7](http://systemsbiology.cau.edu.cn/agriGOv2/termDetail.php?session=944978279.1&GO=GO:0004601) | 159 | 0.0012 | 0.032 |
| GO:0016684 | F | oxidoreductase activity, acting on peroxide as acceptor | [7](http://systemsbiology.cau.edu.cn/agriGOv2/termDetail.php?session=944978279.1&GO=GO:0016684) | 162 | 0.0013 | 0.032 |
| GO:0016301 | F | kinase activity | [26](http://systemsbiology.cau.edu.cn/agriGOv2/termDetail.php?session=944978279.1&GO=GO:0016301) | 1415 | 0.0015 | 0.034 |
| GO:0016209 | F | antioxidant activity | [7](http://systemsbiology.cau.edu.cn/agriGOv2/termDetail.php?session=944978279.1&GO=GO:0016209) | 178 | 0.0022 | 0.046 |
